# Effect of Divalent Metal Ion on the Structure, Stability and Function of *Klebsiella pneumoniae* Nicotinate-Nucleotide Adenylyltransferase: Empirical and Computational Studies

**DOI:** 10.3390/ijms23010116

**Published:** 2021-12-23

**Authors:** Olamide Jeje, Reabetswe Maake, Ruan van Deventer, Veruschka Esau, Emmanuel Amarachi Iwuchukwu, Vanessa Meyer, Thandeka Khoza, Ikechukwu Achilonu

**Affiliations:** 1 Protein Structure-Function Research Unit, School of Molecular and Cell Biology, Faculty of Science, University of the Witwatersrand, Johannesburg 2050, South Africa; olamide.jeje1@students.wits.ac.za (O.J.); 1480907@students.wits.ac.za (R.M.); 1623683@students.wits.ac.za (R.v.D.); 1473414@students.wits.ac.za (V.E.); emmanuel.iwuchukwu@wits.ac.za (E.A.I.); 2 Functional Genomics and Immunogenetics Laboratory, School of Molecular and Cell Biology, Faculty of Science, University of the Witwatersrand, Johannesburg 2050, South Africa; vanessa.meyer@wits.ac.za; 3 Department of Biochemistry, School of Life Sciences, Pietermaritzburg Campus, University of KwaZulu-Natal, Pietermaritzburg 3209, South Africa; khozat1@ukzn.ac.za

**Keywords:** nicotinate nucleotide adenylyltransferase (NNAT), *Klebsiella pneumoniae*, ATP, β nicotinamide mononucleotide, NAD^+^, cations, stability, computational modelling, protein expression, thermal shift assay

## Abstract

The continuous threat of drug-resistant *Klebsiella pneumoniae* justifies identifying novel targets and developing effective antibacterial agents. A potential target is nicotinate nucleotide adenylyltransferase (NNAT), an indispensable enzyme in the biosynthesis of the cell-dependent metabolite, NAD^+^. NNAT catalyses the adenylation of nicotinamide/nicotinate mononucleotide (NMN/NaMN), using ATP to form nicotinamide/nicotinate adenine dinucleotide (NAD^+^/NaAD). In addition, it employs divalent cations for co-substrate binding and catalysis and has a preference for different divalent cations. Here, the biophysical structure of NNAT from *K. pneumoniae* (KpNNAT) and the impact of divalent cations on its activity, conformational stability and substrate-binding are described using experimental and computational approaches. The experimental study was executed using an enzyme-coupled assay, far-UV circular dichroism, extrinsic fluorescence spectroscopy, and thermal shift assays, alongside homology modelling, molecular docking, and molecular dynamic simulation. The structure of KpNNAT revealed a predominately α-helical secondary structure content and a binding site that is partially hydrophobic. Its substrates ATP and NMN share the same binding pocket with similar affinity and exhibit an energetically favourable binding. KpNNAT showed maximum activity and minimal conformational changes with Mg^2+^ as a cofactor compared to Zn^2+^, Cu^2+^ and Ni^2+^. Overall, ATP binding affects KpNNAT dynamics, and the dynamics of ATP binding depend on the presence and type of divalent cation. The data obtained from this study would serve as a basis for further evaluation towards designing structure-based inhibitors with therapeutic potential.

## 1. Introduction

The spike in the rate of nosocomial infection caused by multi-drug resistant (MDR) bacteria is alarming and remains a major global concern to health care systems. As identified by Rice (2008), a group of MDR strains known as ESKAPE pathogens, consisting of both Gram-positive and negative species (*Enterococcus faecium*, *Staphylococcus aureus, Klebsiella pneumoniae, Acinetobacter baumannii, Pseudomonas aeruginosa*, and *Enterobacter* spp.), are the predominant cause of nosocomial infections [1]. These pathogens are notorious for their ability to effectively escape the biocidal action of antibacterial drugs; hence, they are difficult to eradicate [1,2] and contribute to severe infections, prolonged hospitalisation, and death [3].

*K. pneumoniae* accounts for a significant proportion of nosocomial infections, including urinary tract infections, pneumoniae, bloodstream infections, liver abscess, meningitis, wound or surgical site infections, and septicemias [4]. About 7–14% of nosocomial infections in ICU and 3–20% of neonatal septicemia are caused by *K. pneumoniae*. The significant increase in recognition of *K. pneumoniae* as a nosocomial-causing pathogen is attributed to the substantial antibiotic resistance, demonstrated due to their ability to acquire MDR plasmids among different members of the Enterobacteriaceae [4]. This is evident in the resistance of *K. pneumoniae* to extended-spectrum cephalosporin, carbapenem, penicillin, monobactams, extended-spectrum β-lactamases (ESBLs), and fluoroquinolones [3,4]. Though many innovative therapies, including combination therapy, an improvement on β-lactamase inhibitors, and new antibiotics [5] have been developed in recent times to combat multi-resistant *K. pneumoniae*, their insurgence persists [6]. Thus, signifying the urgency to identify novel alternative targets and effective antibacterial agents. Currently, most of the prevailing antibiotics utilise DNA synthesis and cellular functions like interfering with proteins involved in the synthesis of cell wall targets. It is therefore paramount that alternative targets be identified.

Nicotinamide adenine dinucleotide (NAD^+^) has been a major focus of investigation [7,8,9,10] owing to the indispensable roles of NAD^+^ and its derivatives in cell survival. NAD^+^ is essential in driving hundreds of biochemical reactions and metabolic pathways, especially as an energy and signal transducer. Its indispensability revolves around its direct effect on nearly all metabolic pathways, participating in cellular energy metabolism and reductive biosynthesis, and the maintenance of redox activity [11,12]. Furthermore, NAD^+^ is utilised in DNA repair [13], protein modification and gene regulation [14]. Given these increasingly vital roles of NAD^+^ and its derivatives in cellular and biological processes, it is imperative to sustain NAD^+^ homeostasis. An alteration in NAD^+^ synthesis or degradation regulation would likely impair the cell’s metabolic state [15] and ultimately result in death. Hence, the biosynthesis of NAD^+^ appears to be a promising druggable target to explore new therapeutic agents against the infections associated with the MDR strains.

NAD^+^ biosynthesis is accomplished in bacteria by two biochemical pathways: the de novo-synthesis and the pyridine ring-salvage pathway [16]. Both pathway products, nicotinamide/nicotinic acid mononucleotide (NMN/NaMN), link at the reaction catalysed by nicotinate-nucleotide adenylyltransferase (NMNAT/NNAT) to form NAD^+^. The reaction catalysed by NNAT constitutes the crucial step leading to the formation of NAD^+^. It involves the transfer of the adenyl group from ATP to NMN/NaMN, forming NAD^+^ [17] with the release of inorganic pyrophosphate. Since there is no other pathway leading to NAD^+^ formation, the role of NNAT activity in NAD^+^ synthesis is unequivocally indispensable. Furthermore, compared to other enzymes driving the downstream formation of NAD^+^, bacteria NNAT exhibits a minor sequence identity and structural similarity to its human homolog [7,18]. Thus, making it a more suitable antibacterial target for the design of specific inhibitors. Besides, studies on structure-based inhibitor development, using a protein knockdown approach to induce deprivation of NNAT, demonstrated antibacterial activity [9,10,15]. These findings validate NNAT to be an attractive drug target.

The study of NNAT has been widely embraced in different species from archaea, bacteria, and eukaryotes. The respective enzymes exhibit various structural and catalytic properties that reflect their species and specificity. Sequel to the first bacteria NNAT discovered in *E. coli* [19], several bacteria NNAT have been characterised [17,20,21,22,23], and these enzymes utilise either or both nicotinic acid mononucleotide (NaMN) and β nicotinamide mononucleotide (NMN) as substrates, but with a different degree of specificity. Amongst the ESKAPE group of bacteria, *Staphylococcus aureus* [20], *Acinetobacter baumannii* [24], and *Pseudomonas aeruginosa* [17] have been well characterised. A distinctive feature of Gram-positive bacteria NNAT, as seen in *S. aureus* (SaNNAT) [20], *B. anthracis* (BaNNAT) [21], and *B. subtilis* (BsNNAT) [22], are distinguishable by structure dimerisation and substrate preference for NaMN. Likewise, the Gram-negative bacteria share a typical distinctive monomeric structure and preference for NaMN substrate as observed in *P. aeruginosa* (PaNNAT) and *E. coli* NNAT (NadD) [17,23,25]. However, *A. baumannii* NNAT (abNadM) displays an equal substrate preference for NMN/NaMN [24].

NNATs are metal activated enzymes requiring divalent metal cations for the catalysis of the adenylyl transfer reaction. These divalent metals are essential for substrate binding and orientation of nucleotides and assist in catalysis. Asides from the physiologically relevant Mg^2+^, different divalent metals like Ca^2+^, Zn^2+^, and Mn^2+^ are reported to function in phosphoryl transfer and substrate binding, but are not necessarily efficient at substrate turnover or product release [26]. Many studies have established NNAT isozyme to have a distinctive preference for varied divalent metal cations. Furthermore, it is suggested that this variation in metal ion specificity may perhaps impact variation in structure, consequently contributing to the distinctive function and substrate utilisation of the NNAT isozyme. The *E. coli* NNAT (NadR) shows a higher preference for Ni^2+^ and Co^2+^ while displaying substrate specificity for NMN [27]. The hNNAT-1 prefers Zn^2+^ and Ni^2+^ to Mg^2+^ [28], whereas hNNAT-2 and hNNAT-3 attain optimal activity with Mg^2+^, though all isomers do not significantly discriminate between substrates [29,30]. Similarly to hNNAT-1, yeast NNAT-1 displayed maximal activity with Ni^2+^ [31] and exhibits equal efficiency towards NMN/NaMN, whereas yeast NMNAT-2 prefers Co^2+^ [32] and has a higher affinity for NaMN [33].

Following the divergence in the structural and catalytic properties of NNAT from various species, it is paramount to understand the structure-function, mechanism, and dynamic behaviour of this family of enzymes. Until now, minimal information has been established on KpNNAT. This information will enable the design of molecular compounds that can selectively inhibit the activity of the enzyme. Hence, in this study, the over-expression and purification of KpNNAT, its distinctive structural and functional features, and the impact of different divalent metal cations on the activity, conformational dynamics and ligand binding are described. This study was approached using empirical and computational modelling. The data obtained is beneficial for the novel application of the enzyme and as a molecular basis for further evaluation in the design of structure-based inhibitors with therapeutic potential.

## 2. Results

### 2.1. Expression and Purification of Recombinant KpNNAT

**Figure 1:**
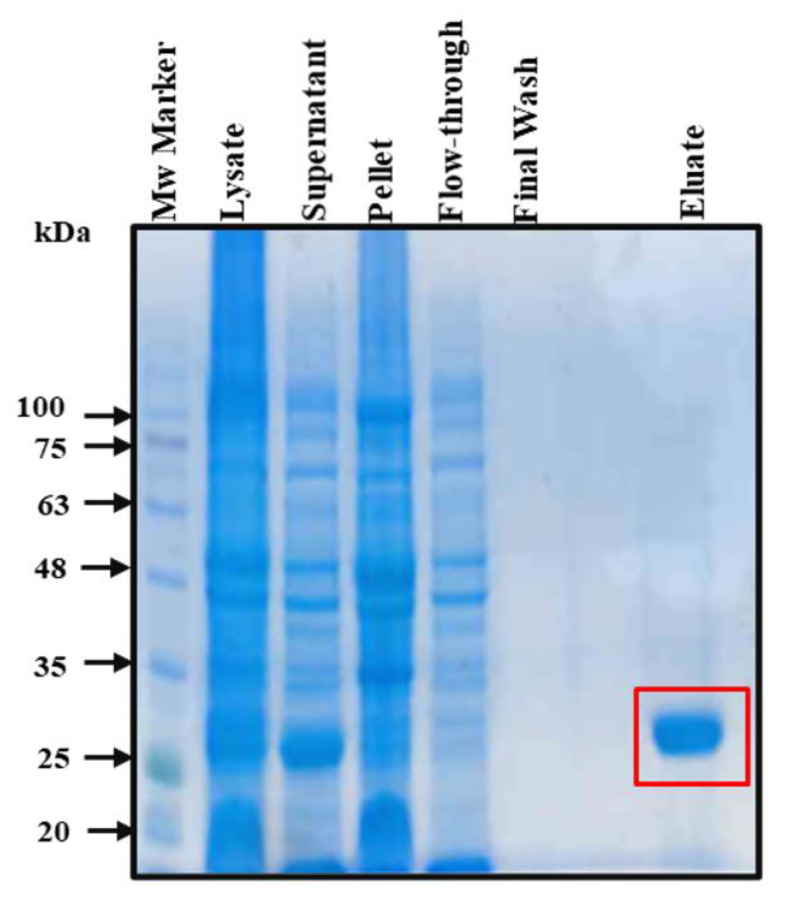
SDS–PAGE analysis of expressed and purified NNAT from *K. pneumoniae*. Crude lysate of *E*. *coli* containing overexpressed KpNNAT was subjected to centrifugation to separate the soluble fraction (supernatant) from the insoluble (pellet). The solubilised fraction was purified by Ni^2+^-IMAC and eluted with 350 mM imidazole. All fractions were analysed on a 12.5% (*w*/*v*) polyacrylamide gel containing 10% (*w*/*v*) SDS and visualised with Coomassie brilliant blue staining. The square indicates the purified protein, which is ~27 kDa.

Recombinant KpNNAT was overexpressed using a pET-28a expression vector (Figure S1) together with the expression conditions 0.5 mM IPTG, 30 °C, and a 6 h induction time. The amount of wet cell pellet obtained for the expression was approximately 7.5 g/L of culture. The SDS-PAGE analysis of the cell lysate showed an excellent yield of soluble protein, which was purified using a one-step immobilised Ni^2+^-affinity chromatography. The purified KpNNAT sample showed more than 95% purity (Figure 1). The protein concentration was estimated spectrophotometrically as ~7.4 mg/mL (~150 mg KpNNAT from one litre *E. coli* T7 Express^TM^ cultured).

### 2.2. Enzyme Activity Assay, Optimal pH, and Effect of Divalent Metal Ions on KpNNAT

Following the successful over-expression and purification of the recombinant KpNNAT, the properties and functionality of the protein were assayed spectrophotometrically using an enzyme coupled assay involving yeast alcohol dehydrogenase (ADH). This assay quantifies the KpNNAT activity based on the production of NADH from NAD^+^. The activity assay shows that KpNNAT exhibits a broad pH optimum between pH 6.0 and 9.5 with a “pseudo” specific activity of 0.2 µmol/min/mg at pH 8.0.

Since NNATs are metal activated enzymes, the requirement for divalent metal ions in catalysis of the adenylyl transfer reaction was assayed. The protein exhibited no NNAT activity in the absence of metal; however, ADH was active (data not shown). Therefore, the effect of selected divalent cations Mg^2+^, Ni^2+^, Ca^2+^, Cu^2+^, and Zn^2+^ on the activity of KpNNAT was evaluated between pH 5.0 and 9.0 (Figure S2A). Mg^2+^ was the most effective cation but can be substituted to a great extent by Ni^2+^, Zn^2+^, and Cu^2+^ supported partial activity at lower pH while Ca^2+^ repressed KpNNAT activity (Table 1).

To eliminate the possibility of protein aggregation which may be induced by metal ion-binding due to the presence of the N-terminal 6× His tag, Rayleigh scattering was carried out using UV-Vis spectrophotometry. The results obtained (Figure S2B) showed that Zn^2+^, Ca^2+^, Mg^2+^, and Ni^2+^ did not induce aggregation of the protein, even at a concentration as high as 5 mM (divalent cations) and 250 µM (KpNNAT).

### 2.3. Far-UV Circular Dichroism Spectroscopy 

**Figure 2:**
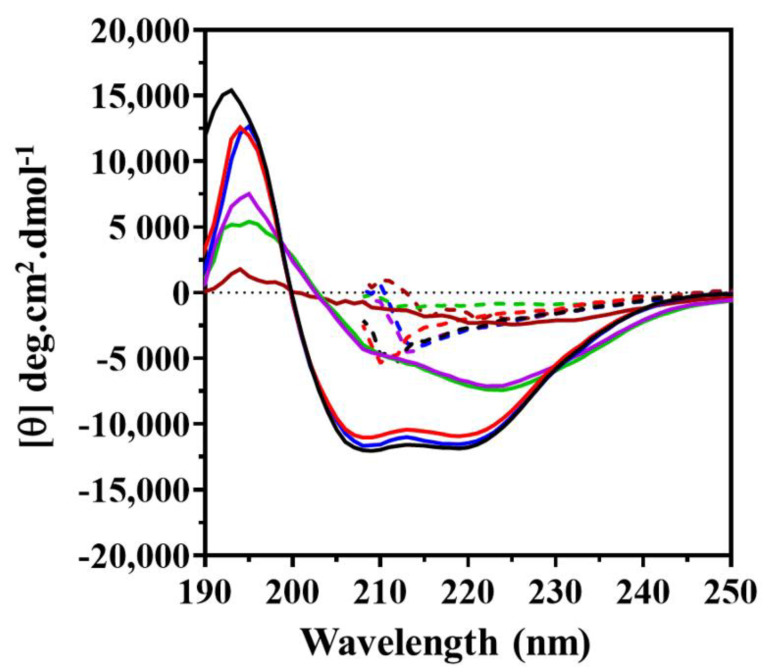
Characteristic far-UV circular dichroism spectra of KpNNAT collected at 20 °C, in the absence of any divalent cation (black lines), and in the presence of Mg^2+^ (blue lines), Ca^2+^ (red lines), Ni^2+^ (purple lines), Cu^2+^ (brown lines) and Zn^2+^ (green lines). The dashed lines represent spectra of the denatured enzyme (in the presence of 8 M urea). The recombinant enzyme (5 µM) was analysed in ddH_2_O using 1 mm path length, 5 nm bandwidth, and 1 nm data pitch. Each spectrum represents an average of three replicates. The plot was generated using GraphPad Prism.

The secondary structure content of KpNNAT was determined using far-UV circular dichroism spectroscopy (CD). The peptide bonds of KpNNAT served as a chromophore in the far-UV region based on their ability to polarise light. The resulting CD spectra (Figure 2) showed a peak of positive ellipticity at 190 ± 0.5 nm and two troughs with a negative ellipticity at 220 ± 0.5 nm and 208 ± 0.5 nm, suggesting that the recombinant KpNNAT protein is predominately α-helical. Further analysis of the secondary structure content in the presence of selected divalent cations showed that in the presence of Mg^2+^ and Ca^2+^, KpNNAT sustained it predominate α-helical structure. In the presence of Ni^2+^ and Zn^2+^, the two negative ellipticity troughs at 220 ± 0.5 nm and 208 ± 0.5 nm were no longer observable, indicating loss of helical content. The presence of Cu^2+^ completely unfolded the protein with no defined secondary structure, which was comparable to the spectra for denatured proteins. The deconvolution of the CD spectra data via DichroWeb analysis program, CONTINLL algorithm, Reference Set 6, further revealed the percentage composition of KpNNAT secondary structure in the absence and presence of the selected divalent cations (Table 2).

### 2.4. ANS Fluorescence Spectroscopy

**Figure 3:**
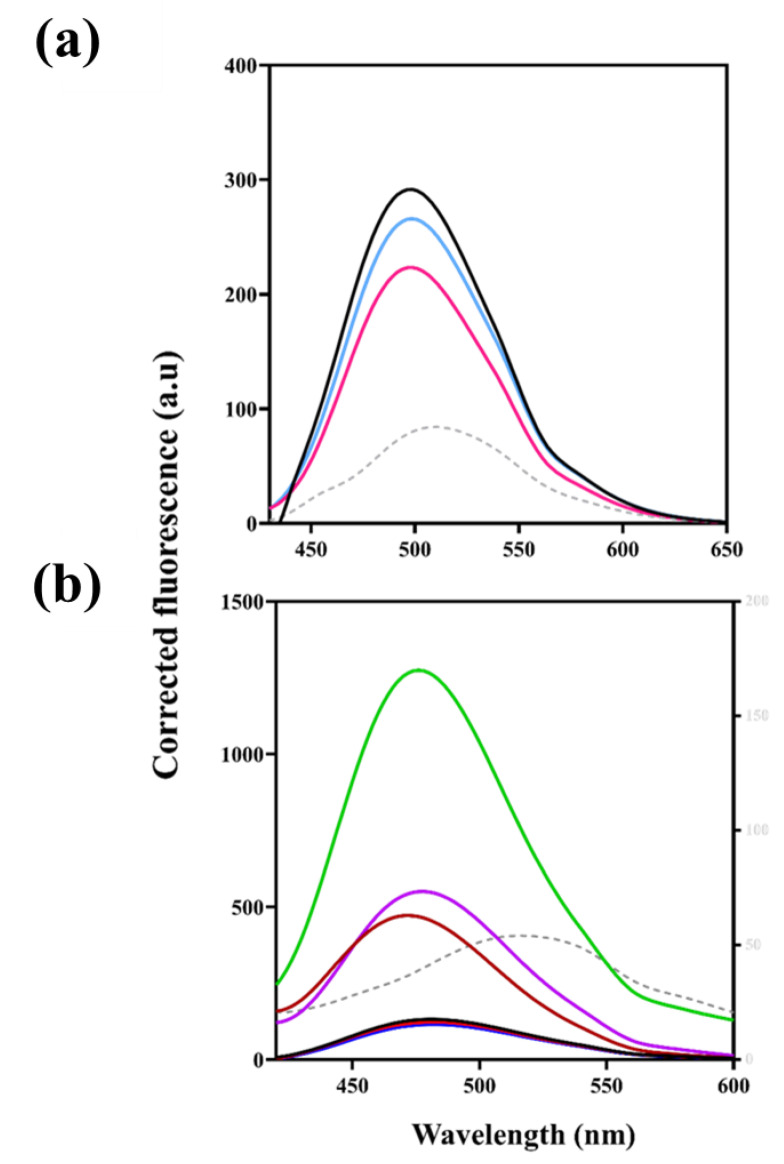
ANS fluorescence emission spectra of KpNNAT. (**a**) in the presence of ATP (pink line), NMN (blue line), and 8 M urea (grey dotted line). (**b**) KpNNAT in the absence of divalent cation (black line), and in the presence of Mg^2+^ (blue line), Ca^2+^ (red line), Ni^2+^ (purple line), Cu^2+^ (brown line) and Zn^2+^ (green line). The grey dotted line represents the average free ANS in the six conditions. All measurements were performed in 25 mM Tris-HCl, 0.01% (*w*/*v*) NaN_3_, pH 7.5 containing 50 µM ANS and 5 µM protein. Excitation was at 395 nm and emission spectra were collected between 400 and 650 nm. The plot was generated using GraphPad Prism.

The hydrophobicity of the recombinant protein binding site and the interaction of the ligands with the protein were analysed using the anionic dye, 8-Anilino-1-naphthalene sulfonic acid (ANS) (Figure 3a). This was possible because of the spectral properties of ANS. Upon excitation of the samples at 395 nm, ANS fluorescence spectra were obtained between 400 and 650 nm emission wavelength. The binding of ANS to KpNNAT yielded a significant increase in fluorescence intensity and a blueshift in the maximum emission wavelength, from 508 nm to 499 nm, which is characteristic of ANS binding to a hydrophobic pocket of a protein. Compared to its native form, the interaction of ANS with denatured KpNNAT yielded a 12 nm redshift and a decrease in fluorescence intensity, suggesting a well-folded protein with a hydrophobic binding pocket. The binding of ATP and NMN to KpNNAT did not show any observable shift in the maximum emission wavelength of ANS. However, a decrease in quantum yield was observed, indicating the displacement of ANS by the substrates (ATP and NMN) for the hydrophobic pocket of the protein and the possibility of ANS and the ligands accessing the same binding pocket.

We further characterised the effect of selected divalent cations on ANS binding spectroscopy. KpNNAT was exposed to either no divalent cation or, Mg^2+^, Ni^2+^, Ca^2+^, Cu^2+^, or Zn^2+^ (Figure 3b). Results suggest that the protein’s overall tertiary structure or local ANS binding environment may be altered by Zn^2+^ and Cu^2+^ and to a lesser extent by Ni^2+^. Hence, there was an increase in quantum yield and a concomitant blue shift in the maximum emission wavelength of the protein. Mg^2+^ and Ca^2+^ did not affect the enzyme’s overall tertiary structure or local ANS binding site.

### 2.5. Mant-ATP Spectroscopy

**Figure 4:**
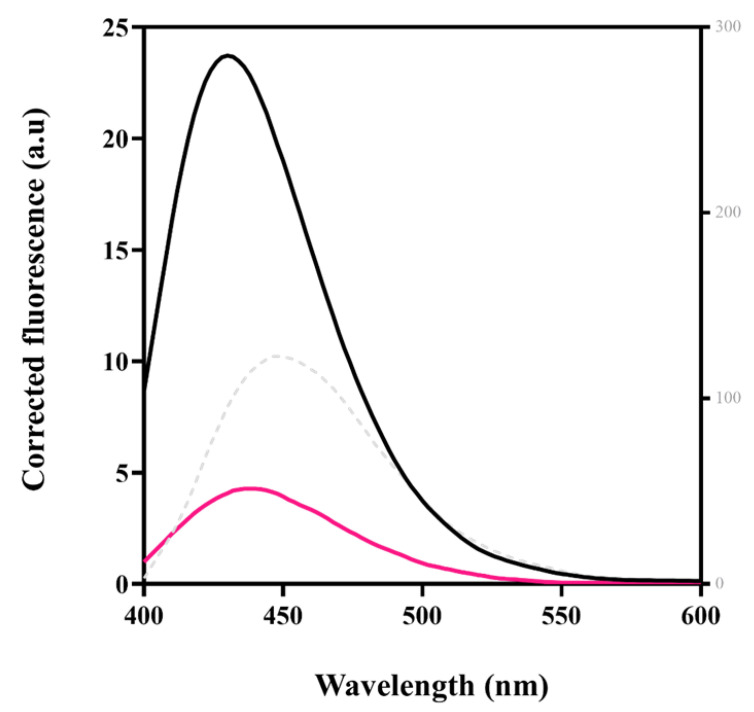
Fluorescence emission spectra of mant-ATP binding to KpNNAT. The binding of mant-ATP to the enzyme alone (black line), the enzyme in the presence of 1 mM ATP (pink line) and in the presence of 8 M urea (grey dotted line). Analyses were performed in 10 mM NaH_2_PO_4_, 0.01% NaN_3_, pH 7.5 containing 10 µM mant-ATP and 2.5 µM protein. Excitation was at 355 nm and emission spectra were collected between 400 and 600 nm. The plot was generated using GraphPad Prism.

The proclivity of KpNNAT to bind ATP was measured using mant-ATP extrinsic fluorescence (Figure 4). Mant-ATP was excited at 355 nm and its emission spectra collected between 400 and 600 nm. The interaction between mant-ATP and KpNNAT was accompanied by an increase in quantum yield and a blue shift in maximum emission wavelength from 450 to 430 nm, which is characteristic of mant-ATP binding to nucleotide-binding sites. The binding of ATP decreased the fluorescence of mant-ATP, indicating that ATP must have outcompeted mant-ATP for the same binding pocket. When the enzyme was denatured in the presence of 8 M urea, the overall quantum yield decreased with an 18 nm redshift relative to the native protein (in the absence of ATP), which indicates that ATP binds to a partially hydrophobic specific pocket.

### 2.6. The Effect of Divalent Metals and ATP Binding on the Stability of KpNNAT

**Figure 5:**
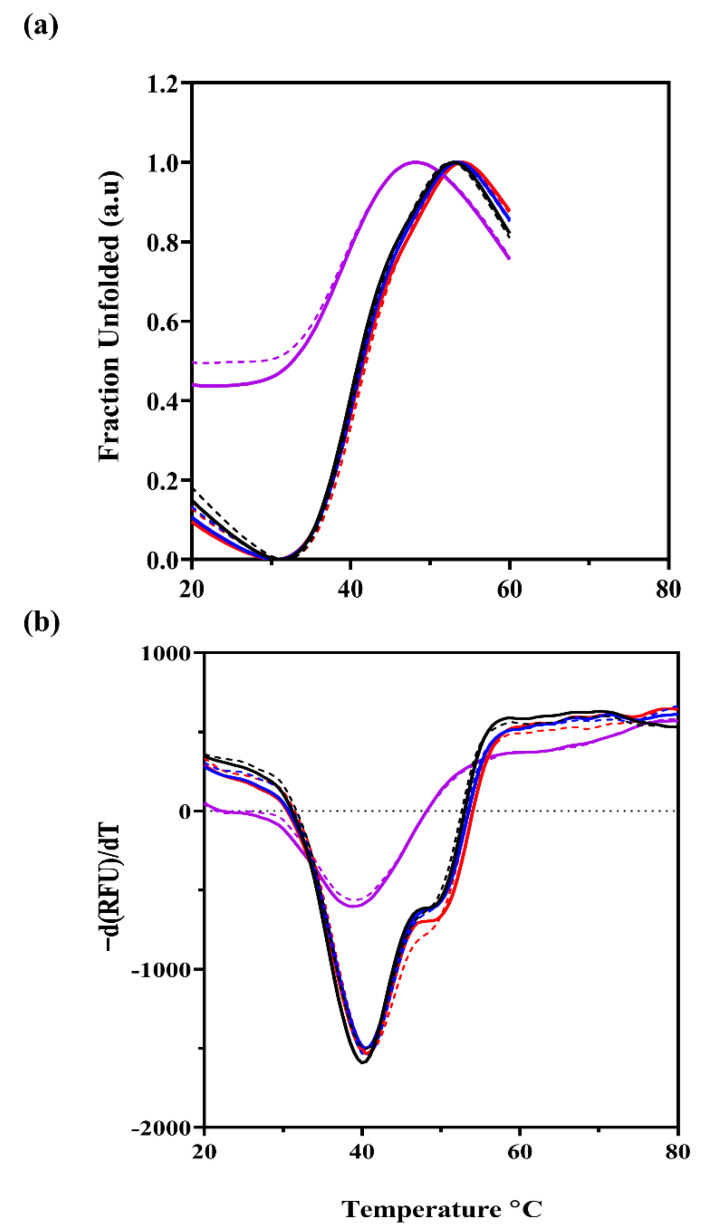
The fluorescence thermal shift analysis of recombinant KpNNAT (**a**) showing the thermal unfolding curves and (**b**) the melting curves of the protein in the absence of divalent cation (black line), and in the presence of Mg^2+^ (blue line), Ca^2+^ (red line), and Ni^2+^ (purple line). The dotted lines indicate the absence of ATP, while the solid lines indicate the presence of ATP. Stability was monitored between 20 °C and 80 °C using SYPRO Orange dye in 25 mM Tris-HCl, pH 7.5 with 0.1 mM ATP and 5 mM salts (MgCl_2_, NiSO_4_, CaCl_2_, ZnSO_4_, and CuSO_4_). The plot was generated using GraphPad Prism.

A fluorescence thermal shift assay was used to study the impact of divalent cations and ATP binding on the stability of KpNNAT (Figure 5). The assay was monitored between 20 °C and 80 °C. The T_m_ of KpNNAT was determined to be 40 °C; however, upon ATP binding, the T_m_ increased by 0.5 °C (Table 3). In the presence of Mg^2+^ and Ca^2+^, the T_m_ of KpNNAT was 40.5 °C, but a marginally higher T_m_ of 41 °C was observed for Ca^2+^ in the absence of ATP. Ni^2+^ demonstrated a high initial unfolding transition and a slight decrease in the T_m_ of KpNNAT, suggesting that the protein was partially unfolded. However, Zn^2+^ and Cu^2+^ appeared to unfold the protein at room temperature, as shown by the lack of a transition in the unfolding curves and high initial RFU values (Figure S3). In all, the stability of KpNNAT appears not to be significantly affected by ATP binding because the T_m_ of KpNNAT-apo is comparable to KpNNAT-ATP complexes.

### 2.7. Molecular Modelling

A homology model of KpNNAT was generated using the *E. coli* NNAT (EcNNAT) homologue (PDB ID: 1K4K) as a template for the Swiss modelling of the KpNNAT model. The 1K4K coordinates show four monomeric units of the enzyme in a single asymmetric unit. However, only one of the monomeric units was used to generate the KpNNAT model. All 209 of the 212 amino acid residues were included in the model, which showed a QMean (Qualitative Model Energy Analysis) score of 0.16 and a GMQE (Global Model Quality Estimation) score of 0.93 (Figure S4), indicating a highly reliable model. The KpNNAT and EcNNAT share 76.42% sequence identity, making the 1K4K coordinate a preferable template for modelling KpNNAT. The backbone Cα root-mean-square deviation between the KpNNAT model and 1K4K was 0.078 Å, indicating a highly comparable model. The structural alignment (Figure S4) between KpNNAT and EcNNAT shows a very reliable structure match between these enzymes. The Ramachandran analysis (Table 4) showed that most residues are in the most favourable regions; no amino acid residues (other than glycine and proline) were in the additionally allowed and disallowed regions. 

The KpNNAT model was subjected to energy minimisation protocol using the OPLS_2005 force field to relax the model and prepare it for subsequent molecular modelling studies. No perturbation was observed with the model upon energy minimisation. The docking of the ligands (ATP and NMN) into KpNNAT using the induced-fit ligand docking protocol implemented in Schrödinger Maestro v12 generated over 14 poses, with an average Emodel score of −98.23 kcal/mol and −77.38 kcal/mol and a Glide Energy of −66.14 kcal/mol and −50.64 kcal/mol for KpNNAT-ATP and KpNNAT-NMN, respectively; projecting that the protein-ligand interaction proceeds exergonically. Overall, the poses revealed that ATP and NMN bind on the same site within the KpNNAT receptor and are stabilised mostly through van der Waals and H-bonding (Figure S6).

The top-scoring poses and the apo-structures were further submitted to the Desmond molecular dynamics simulation (implemented in Maestro v12). The average slopes for total energies, potential energies, and temperature were ±0.005 unit/ps in all systems, indicating that the systems were fully equilibrated before the 100 ns simulations (Table 5).

The trajectories corresponding to the first 20 ns were further submitted to Amber 18 for MM/GBSA binding free energy calculations. The result showed that the binding of ATP and NMN to the receptor is energetically favourable and occurs spontaneously, as demonstrated by the negative ∆*G*_bind_ (Table 6). The per residue energy decomposition plot (Figure S6) showed that both substrates are stabilised by a combination of van der Waals and electrostatic interactions. The stabilisation of ATP within the receptor involves more amino acid side chains than NMN, a relatively smaller molecule.

**Figure 6:**
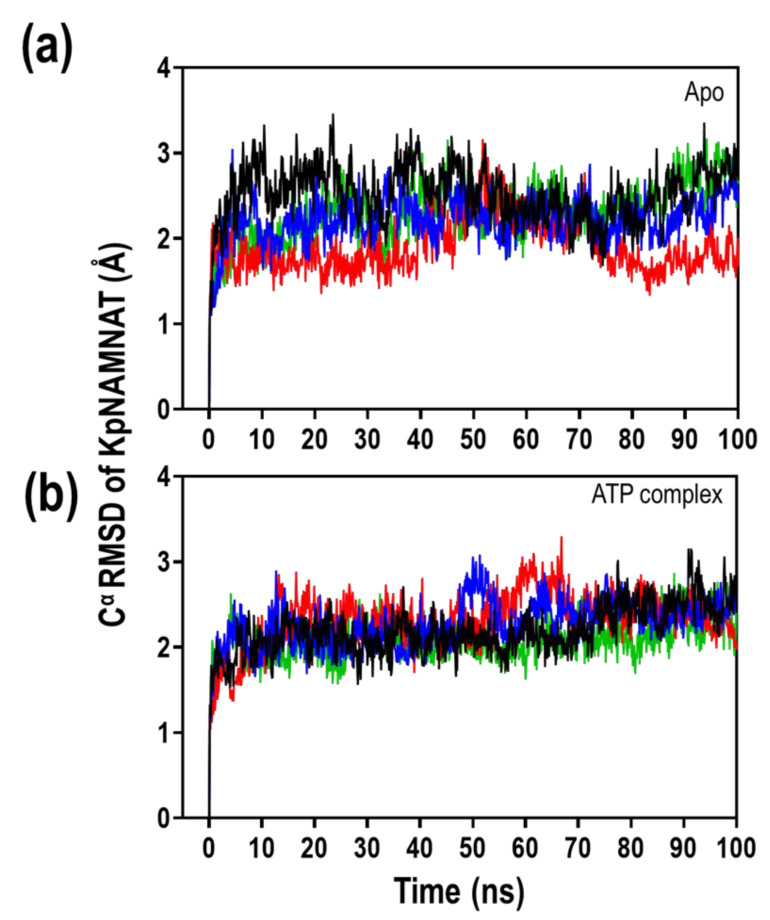
The root-mean-square deviation (RMSD) of the Cα atoms of KpNNAT over 100 ns simulation time. Analysis of KpNNAT apo and KpNNAT-ATP complex in the absence (black) and presence of Mg^2+^ (blue), Ca^2+^ (red), and Zn^2+^ (green). The fluctuation change for all systems is within 3 Å and stabilises within a fixed value at the end of the simulation. The plots were generated using GraphPad Prism.

We further explored the impact of both ATP-binding and three divalent cations on the dynamics of the enzyme and ATP. This was gauged based on the RMSD, the RMSF, and the radius of gyration of both the receptor and the ligand with respect to the receptor. The Cα RMSD of the protein, along the 100 ns simulation time, varied in the presence of the three divalent cations (Mg^2+^, Ca^2+^ and Zn^2+^), with respect to no divalent cations (Figure 6). The addition of ATP slightly reduced the variation in the Cα RMSD of the protein along the 100 ns simulation period. Overall, KpNNAT apo demonstrated the highest Cα RMSD value (Figure S7). At the same time, the presence of ligands (ATP and or metals) stabilises the dynamism of the protein as indicated by the slightly lower Cα RMSD observed.

The changes in the Cα of the protein side chains were characterised using the Cα RMSF. The results obtained exhibited slight variation in the movement of the Cα atoms (Figure S8). In the presence of Mg^2+^ and Zn^2+^, the most fluctuated regions of the protein were between residues 40 and 50; however, upon ATP binding, the fluctuation of this region appeared reduced while the region’s 112–120 and 129–135 residues fluctuated the most in Mg^2+^. Conversely, the regions 112–120 and 129–135 of the protein were observed to fluctuate the most in the presence of Ca^2+^, but these regions became less fluctuated upon ATP binding. This suggests that the protein dynamics are influenced mainly by the type of divalent cation and the presence of ATP in the receptor. 

**Figure 7:**
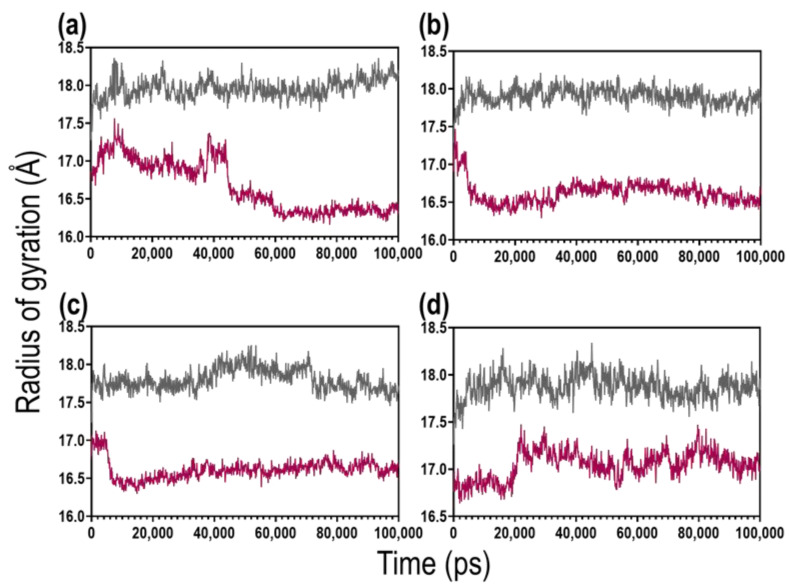
The radius of gyration (Rg) of the Cα atoms of KpNNAT. KpNNAT apo (gray) and KpNNAT-ATP complex (Purple) were analysed with (**a**) no metal (**b**) Mg^2+^ (**c**) Ca^2+^ and (**d**) Zn^2+^ over 100 ns simulation time. The Rg of KpNNAT apo systems are comparable amongst the different metals ions. However, a decrease in the Rg of the protein was observed upon ATP binding. The plots were generated using GraphPad Prism.

The radius of gyration (Rg), which measures the compactness of the protein, showed (Figure 7) that the presence of ATP in the receptor site decreases the protein’s overall Rg (increases compactness). This is also influenced by the type of divalent cation present in the solution. Mg^2+^ and Ca^2+^ decreased the protein’s Rg from 17 Å to 16.5 Å after approximately 10 ns, whereas the presence of Zn^2+^ in the solution increased the Rg (reduces the compactness) of the protein compared to the presence of Mg^2+^ and Ca^2+^.

**Figure 8:**
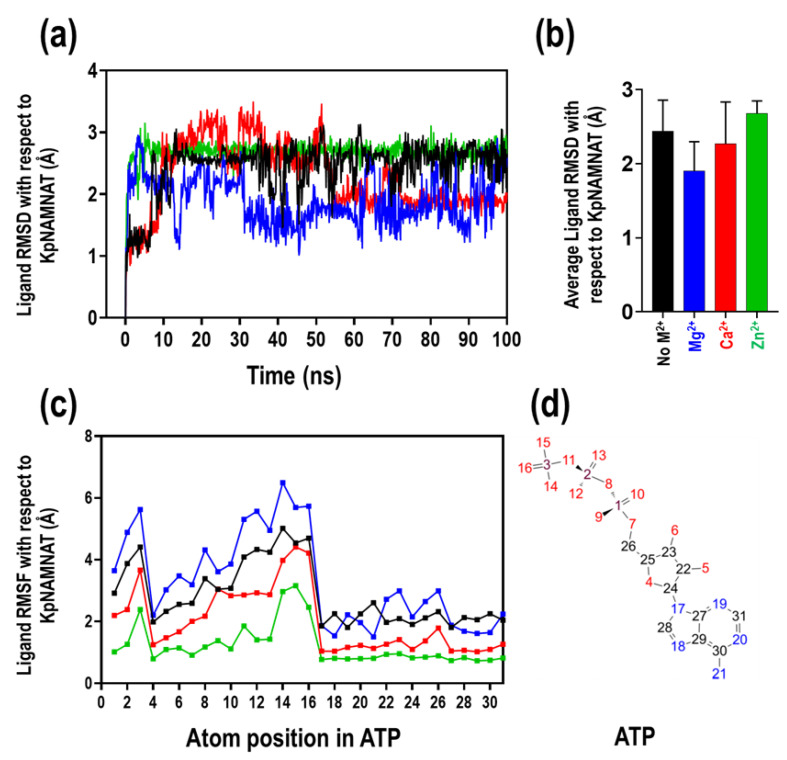
Trajectory analysis showing (**a**) the RMSD of ATP with respect to KpNNAT (**b**) the RMSF of ATP with respect to KpNNAT (**c**) the average RMSD of ATP with respect to KpNNAT in the absence of metal ion (black) and presence of Mg^2+^ (blue), Ca^2+^ (red), and Zn^2+^ (green). The plots were generated using GraphPad Prism. (**d**) Numbering of the ATP molecule in relationship with (**c**). The highly fluctuating atoms of ATP are indicated in red. Image was generated from Schrödinger Maestro 2D diagram.

The conformational dynamics of ATP within the KpNNAT binding site and its contacts with the protein and the environment was investigated in the absence and presence of metals, using the ligand RMSD, the ligand RMSF and the protein side-chain interaction plots over a 100 ns MD simulation. The ligand RMSD of the KpNNAT-ATP complex systems showed that Zn^2+^ steadily stabilises the ligand throughout the trajectories, comparable to when there is no metal (Figure 8a). In the presence of Ca^2+^, the ligand demonstrates increasing fluctuation compared to Zn^2+^ and no metal. This fluctuation was then stabilised within the binding site in the last 45 ns of the simulation time. The most fluctuation was observed in the presence of Mg^2+^, indicating that the ligand is highly dynamic in Mg^2+^ compared to Zn^2+^ and Ca^2+^. Despite the fluctuation, the ligand did not diffuse or drift away from the binding pocket, as shown by the lower average ligand RMSD observed for Mg^2+^ with respect to the protein complex in the absence of metal (Figure 8b).

Using the RMSF of individual atoms in the ligand, with respect to the protein receptor, the interaction of the ATP ligand with the KpNNAT’s entropic role in the binding event was monitored (Figure 8c). From the entire ATP structure (Figure 8d), the atoms numbered in red representing the phosphate region are the most dynamic part of the ligand. This region fluctuated the most in the presence of Mg^2+^ compared to Ca^2+^, Zn^2+^, and in the absence of metal. The fluctuation observed for Ca^2+^ and Zn^2+^ in this region is much lower than when there is no metal, signifying that the regions are less dynamic; hence, the ligand is more stable in the protein receptor.

**Figure 9:**
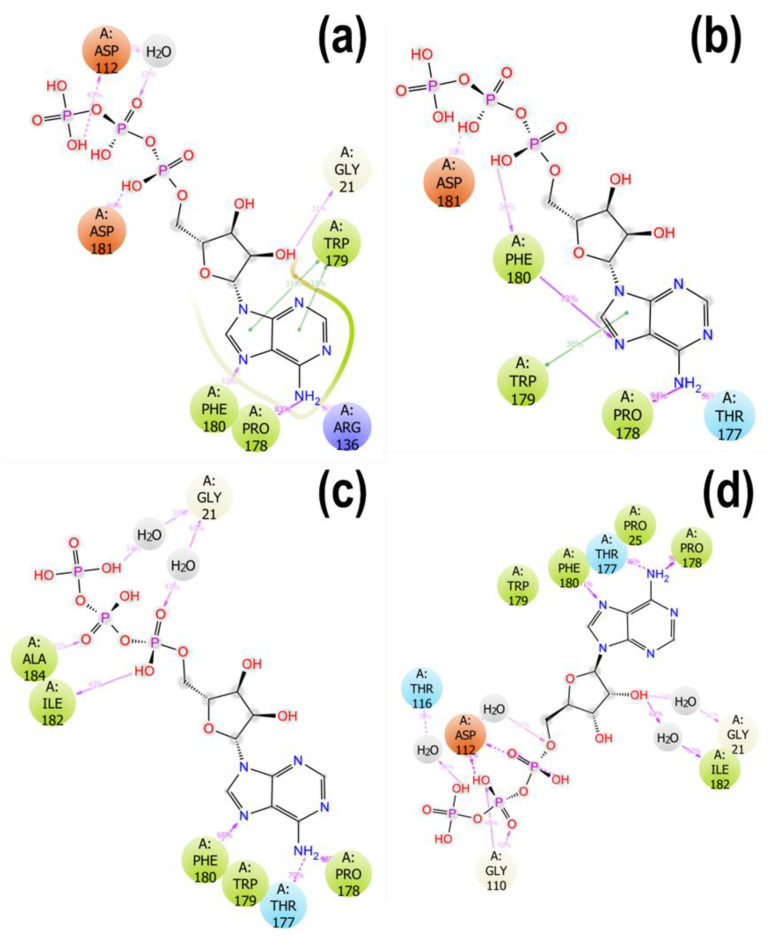
2D interaction plot showing KpNNAT-ATP interactions that occur for more than 30.0% of the 100 ns simulation time for (**a**) no metal (**b**) Mg^2+^ (**c**) Ca^2+^ and (**d**) Zn^2+^.

Detailed ATP atom interactions with the protein residues were obtained using the ligand-protein interactions that occur for more than 30.0% of the 100 ns simulation time. ATP made the most contact with the protein side chains in the presence of Zn^2+^ compared to Ca^2+^, Mg^2+^, and no metal (Figure 9). The presence of residues such as Asp112 making contacts of about 93% with ATP and other side chains, as observed in the presence of Zn^2+^, allowed for tighter binding of ATP to the protein compared to the other metals. Additionally, the stacked bar charts of side-chain interactions between ATP and KpNNAT (Figure S9) showed that the highest interaction fraction of ATP with Asp112 residue was observed in the presence of Zn^2+^, followed by no metal, and then Ca^2+^. Mg^2+^ displayed the lowest interaction fraction between ATP and Asp112. In the presence of Mg^2+^, ATP made contact with fewer residues, most of which were with Pro178, Phe180, and Thr177, allowing more fluctuation within the protein receptor. Analysis of the data obtained from the trajectory cluster (Figure S9) and stacked bar charts of side-chain interactions between ATP and KpNNAT (Figure S10) showed that ATP is stabilised through a combination of salt bridges, hydrogen bonds, ionic contact, hydrophobic interaction, and water molecules within 4 Å of the ligand. In the presence of Mg^2+^, ATP attracted more water molecules (11 molecules) than Ca^2+^ and Zn^2+^, which attracted eight water molecules.

In contrast, six water molecules were attracted to the binding interface without metal ion (Figure S9). An interface with lesser or no water molecules between the ligand and the protein may yield a higher binding affinity than a ligand-binding interface occupied by water molecules. With respect to the fraction of interaction stabilising ATP (Figure S9), more salt bridges and ionic contacts were made by ATP in the presence of Ca^2+^, compared to Mg^2+^. These contacts are very tight binding; hence, stabilising the ligand more in the receptor than Mg^2+^. The ion Zn^2+^, on the other hand, showed the highest fraction of binding, indicating that more interactions are involved in stabilising the ligand and will be more tightly bound than the presence of Mg^2+^.

## 3. Discussion

Understanding the biophysical structure of an enzyme is paramount to its novel application and the design of inhibitors with therapeutic potential. In this study, we have comprehensively described the biophysical features of recombinant KpNNAT in relation to the effect of divalent cations on the structure and function of this essential enzyme. Soluble KpNNAT was over-expressed in *E. coli* and purified to homogeneity using single-step immobilised-Ni^2+^ affinity chromatography. This is evident by the result obtained from the expression profile of the recombinant protein (Figure 1), as the band signifying the presence of the proteins on the SDS-PAGE agrees with the theoretical molecular weight (~27 kDa), deduced using the ProtParam algorithm [34] for KpNNAT. Equally, the one-step affinity purification using Ni^2+^-IMAC, as indicated by the SDS gel, effectively isolated pure His tagged KpNNAT with a yield of ~50 mg of purified KpNNAT per gram of wet cells. Our expression study shows that KpNNAT can be over-expressed and purified to homogeneity, essential for structure-function studies, including X-ray crystallography.

We used the pET28a vector, containing two optional His tags at both ends (N- and C-terminus). However, we designed the vector with a stop codon preceding the 3’ end restriction endonuclease sequence (*Bam*HI) used for cloning the ORF of KpNNAT (Figure S1). The N-terminal His tag is cleavable, which means we could cleave the N-terminus 6× His tag using thrombin. However, we decided to leave the 6× His tag for two major reasons, (i) the presence of the His tag at the N-terminus stabilised the recombinant protein, even in very high concentrations (>10 mg/mL); this was important for us because we needed such high concentrations for subsequent structural studies, including X-ray crystallisation and Small-Angle X-ray scattering. (ii) The presence of the tag did not affect the catalytic activity of the protein compared to another construct of KpNNAT we designed using pGEX-4T1. Instead, KpNNAT, expressed using pGEX-4T1 (currently under review, published in Protein Journal), tend to aggregate after thrombin cleavage of the GST-tag, even in the presence of 10% glycerol and 5 mM DTT.

Having obtained high quantities of the pure enzyme, it was important to show that our enzyme was active. The predicted activity of KpNNAT was further confirmed using a dual enzyme-catalysed reaction involving ADH [35]. Due to the involvement of ADH in the assay, the activity calculated can be referred to as a “pseudo” specific activity. This is a simple approach because direct assay would not have been possible for a steady-state kinetic response such as this. After all, the product (NAD^+^) cannot be quantified in real-time without converting to NADH, which has maximum absorption at 340 nm. Nonetheless, it must be noted that without NAD^+^, no NADH will be produced in the assay. Therefore, all NADH quantified in the coupled enzyme assay depended on the NAD^+^ produced by the KpNNAT-catalysed reaction. 

Generally, NNATs are globular proteins belonging to the α/β class. The N-terminus of NNATs is characterised by the presence of α-helices (α), and β-strands (β), whereas the C terminal domain of NNATs mainly comprises of α-helices and loops [33]. In bacterial NNAT, this C-terminus domain functions in the identification of substrate preference. From the far-UV CD conducted, we were able to show that the secondary structure content of KpNNAT is predominantly α-helical by the presence of a distinctive positive peak at 190 nm and two troughs with a negative ellipticity at 220 nm and 208 nm (Figure 2). We further estimated the percentage composition of the secondary structure features of the protein using an online server that analyses the CD spectra via ridge regression analysis to fit the spectrum of the protein by comparing it to the spectra of an extensive database of proteins characterised by X-ray crystallography [36]. The data obtained from the deconvolution of the CD spectra showed the secondary structure composition of KpNNAT as 36.3% α-helix, 16.9% β-strand, 24.4% β turn, and 22.5% unordered, which is comparable to the homology model (Figure S4) and typical of most identified NNATs [17,23,37,38,39]. This is no surprise considering that all NNAT share a common nucleotide-binding domain characterised by alternating α-helices and β-strands. Several helices are imperative for catalysis and play a role in substrate interactions [23].

Because 6× His tag binds divalent cations such as Zn^2+^, Ni^2+^, Co^2+^, and Cu^2+^, we carried out Rayleigh scattering (Figure S2B). Our results suggest that Zn^2+^, Ca^2+^, Mg^2+^, and Ni^2+^ did not result in aggregation of the protein. Instead, Zn^2+^ partially unfolded the protein without aggregating the protein. This corroborates our spectroscopy, far-UV CD, and thermal shift assay studies, which all showed that Zn^2+^ and Cu^2+^ unfolded the KpNNAT, while Ca^2+^, Mg^2+^, and Ni^2+^ did not induce severe changes in the global structure of the protein. Therefore, if the His tag resulted in aggregation of the protein, especially in the presence of Ni^2+^ (i) the enzyme would have lost its activity in the presence of Ni^2+^, (ii) the absorbance at 340 nm would have been high in comparison with the absorbance at 280 nm, (iii) the transparency of the sample used for far-UV CD would have been low and hence, no meaningful data would have been derived from such a sample. The loss in ellipticity troughs (minima) at 220 ± 0.5 nm and 208 ± 0.5 nm suggests a loss in helical contents in the presence of these two metals, even though the protein maintained residual enzyme activity (at pH 7.0–9.0) in the presence of Ni^2+^, but completely lost enzyme activity in the presence of Zn^2+^. This underscores the significance of this study, which led us to postulate that the conformational dynamics of KpNNAT (especially global conformations and local ATP-binding site conformations) may be affected by these divalent cations. This may not have been due to aggregation because the transparency of the sample was high enough that there was a high signal-to-noise ratio, which permitted the observation of ellipticity maxima at 195 nm, unlike the spectrum of KpNNAT in the presence of Cu^2+^, which suggests global unfolding of KpNNAT.

Insight into the tertiary structure and ligand binding properties of the recombinant protein was probed using ANS and mant-ATP, owing to their strong fluorescence energy transfer properties. The interaction of these extrinsic fluorescent dyes with the hydrophobic pockets (ANS) or nucleotide-binding site (mant-ATP) on a protein is often accompanied by a blueshift in the maximum emission wavelength and an increase in fluorescence intensity or quantum yield [40,41]. Following the observable attributes of these fluorescent dyes, a blueshift in maximum emission wavelength coupled with an increase of fluorescence intensity (quantum yield) was observed upon the binding of ANS or mant-ATP to KpNNAT, thus establishing the presence of binding pocket. This binding pocket appears hydrophobic, as indicated by the shifts of 12 nm and 18 nm in the ANS and mant-ATP spectroscopic studies. This is in addition to the significant difference seen in the overall quantum yield (Figure 3A).

Metal ions serve many functions in proteins, including structural stability, enhancement of the proteins in the active conformation required for biological function, modification of protein structures, and regulation or catalytic processes of enzymes. The presence of divalent cations such as Mg^2+^ is crucial for activity in ATP-binding proteins because they participate in substrate-binding and the orientation of nucleotides and assist in catalysis. In NNAT, Mg^2+^ is presumed to polarise the active site, thereby facilitating the nucleophilic attack on the α- phosphorous of ATP [42]. The destabilisation of the ester bond between the α- and β-phosphate of ATP displaces the adenyl group, which is transferred to NMN [43]. Asides from the physiologically relevant Mg^2+^, many studies have also established the NNAT isozyme to have a distinctive preference for varied divalent metal cations. KpNNAT attained maximal activity in the presence of Mg^2+^ and, to a substantial extent, Ni^2+^, while its activity was suppressed in the presence of Ca^2+^, Cu^2+^, and Zn^2+^ (Figure S2A).

Thermal shift assay was used to assay the effect of ATP-binding and divalent cations on the conformation and stability of KpNNAT, alongside far-UV CD and ANS fluorescence spectroscopy. When a ligand binds to protein, they tend to rigidify proteins, consequently increasing their stability. This attribute can be identified by an increase in thermal stability indicated by a rightward shift in the unfolding transition and an increase in the *T*_m_. Upon the binding of ATP to KpNNAT, the changes observed in the thermal stability were minimal. There was no visible shift in the unfolding curve (Figure 5); however, an increment of about 0.5 °C was observed in the *T*_m_ (Table 3), suggesting that ATP binding does not significantly impact KpNNAT stability.

Similarly, Mg^2+^ and Ca^2+^ appear not to perturb the stability of KpNNAT, as indicated from the unfolding transition curve and the 0.5 °C *T*_m_ change compared to the apo form. This was also evident in the secondary-structure content analysis, which showed that the presence of Mg^2+^ and Ca^2+^ did not significantly impact the secondary structure of the protein (Figure 2). Although changes in the polarity of the binding site were observed from the ANS studies, this is no surprise, given that the binding of metal cations should polarise the active site to allow for substrate binding and adenylyl transfer [42]; thus, indicating their efficiency in stabilising the NNAT-substrate complex. As shown in previous studies, not all metals that substitute Mg^2+^ in substrate binding and phosphoryl transfer are efficient at substrate turnover [26,44]. This is possibly true for Ca^2+^, given that its presence suppresses KpNNAT activity despite demonstrating similar attributes as Mg^2+^ in the far-UV CD and fluorescence assays.

The presence of Zn^2+^, Cu^2+^ and Ni^2+^ alters the conformation of KpNNAT. This is evident from the loss of helical content and the subsequent increase in the percentage of unordered structures observed for Ni^2+^, Zn^2+^, and Cu^2+^ (Figure 2 and Table 2). The lack of unfolding transition observed for KpNNAT in the presence of Zn^2+^ and Cu^2+^ (Figure S2A), as well as the high initial unfolding transition shown by Ni^2+^ (Figure 5), not only confirmed that Zn^2+^, Cu^2+^, and Ni^2+^ alters the conformation of KpNNAT but also validates that the type of structure in the subunit interfaces and factors, such as a decrease in the number of hydrogen bonds, helical propensity, ion-pairs, salt bridges, and van der Waals contacts, impact protein stability and contributes to the magnitude of conformational stability [45,46,47]. At room temperature, Zn^2+^ completely unfolds KpNNAT, while Cu^2+^ unfolds about 70% of the protein. Ni^2+^ only demonstrates partial unfolding of the protein. This unfolding of KpNNAT exposes its hydrophobic pocket, allowing the ANS anionic dye to access the hydrophobic regions and increase fluorescence intensity. Hence, the blueshift and the extremely high quantum yield observed in the ANS binding studies in the presence of Zn^2+^ and the subsequent high yields for Cu^2+^ and Ni^2+^ compared to KpNNAT apo or KpNNAT in the presence of Mg^2+^. The immense conformational changes observed for KpNNAT, in the presence of Zn^2+^ and Cu^2+^, possibly play a role in the poor utilisation of these divalent cations as an efficient cofactor, compared to Ni^2+^ only, which showed a minimal conformational change in the structure of the protein. Moreover, the structural change induced by Ni^2+^ possibly enhances protein movement in the active conformation required for biological function.

Computational modelling has proven to be a helpful tool in describing and complementing experimental studies. Its predictive power provides insight and information about certain data that are sometimes inaccessible from the experiment. In a bid to complement our empirical observation and provide further insight into the biophysical behaviour of recombinant KpNNAT at the atomic level, computational modelling was employed in this study. In the absence of a KpNNAT crystal structure, the interaction of the ligands within the putative ligandin site of the protein receptor cannot be validated. Therefore, homology modelling was carried out to build a 3D model of the protein. This was generated using the *E. coli* NNAT (EcNNAT) homologue (PDB ID: 1K4K), which shares 76.4% sequence identity with KpNNAT as a template. We validated the reliability of the generated 3D model as shown in Figure S4 via Ramachandran plot statistics, which showed that 91.8% of the side chains are in the most favourable regions. As derived from the analysis of 118 structures of an R-factor of no more than 20.0 and maximum resolution of 2.0 Å, a quality model is expected to have more than 90% residue in the most favoured regions [48]. Furthermore, the sequence alignment between KpNNAT and EcNNAT showed that their amino acid residues are highly conserved and exhibit a highly comparable structure, as derived from the Cα RMSD (0.078 Å) of the proteins, which showed a very low deviation in structure when aligned (Figure S4). Taking together these findings, the quality of the modelled 3D structure used in this study was validated and ascertained as reliable to make rational inferences.

As proposed by Bathke et al. [49], while characterising the structure and function of Plasmodium falciparum NNAT (PfNNAT), which was found to be more like its bacterial homologs than its human counterparts, the catalysis of ATP and NaMN (or NMN) occurs through the proximity of the phosphate of NaMN (or NMN) to the α-phosphate of ATP. Based on the induced-fit docking (IFD) between the 3D model and the substrates (ATP and NMN), we could deduce from the three-dimensional interaction plot of the top poses corresponding to the protein-substrate complexes (Figure S5) that ATP and NMN bind at the same active site because no other site in the KpNNAT protein attracted the substrates. This is also supported by the presence of the “H/TXGH” motif (His19, Gly21, and Hie22) at both substrate complexes, which are required for recognition and binding of substrates in NNAT [42].

From the 2D interaction plot of the protein-substrate complexes (Figure S5), it is apparent that the interaction of the side chain residues with the phosphate group of the substrates as observed between ATP and residues Arg137, Asp112 and Ile182, and NMN with residues Thr14, Phe15, and Hie22, made the binding of the anionic dyes such as ANS and mant-ATP less favourable; hence, the displacement of the fluorescent dyes and the concomitant decrease in their fluorescence (Figure 3). Furthermore, the propensity of KpNNAT to bind ATP as measured using mant-ATP not only showed the displacement of mant-ATP but also demonstrates changes in the hydrophobicity of the KpNNAT active site, which can be attributed to the formation of hydrogen bonds between the γ-phosphate group of ATPs with Arg137 and Asp112, and the N6 amino group of the adenine ring with Ile108 and Gly12, thus attracting water molecules to the binding site as well as increasing exposure to solvent. The presence of water molecules at the active site is suggested to play a vital role in recognising ligands, conformational dynamics, stabilisation of protein-ligand complex [50,51] and may contribute to the binding free energy protein-ligand interaction.

Given the possibility of ATP and NMN sharing the same binding site, it is important to determine the binding free energy of the two substrates to KpNNAT to gain insight into the type of interactions that occur during the binding event, the binding affinity of the substrates for the protein receptor, and possibly the kind of binding mechanism that maybe exhibited by the enzyme. Using the MM/GBSA algorithm, which computes binding free energy based on the number of contacts and the type of contacts made by the residues with the ligand [52], the relative binding free energy of ATP and NMN to KpNNAT was calculated. From the result presented in Table 6, we see that the binding of the substrates proceeds spontaneously, as indicated by a negative ∆*G*_bind_. Aside from the gas phase energy, which appeared to be the major contributor of the overall ∆Gbind, offset by the solvation free energy, the binding of ATP and NMN to KpNNAT is largely driven by electrostatic interaction than van der Waals forces. The extremely high electrostatic interaction demonstrated by the KpNNAT-NMN complex is noteworthy, which was almost double that of the KpNNAT-ATP complex.

As derived from the per-residue decomposition analysis (Figure S6), the key residues contributing to this high electrostatic interaction exhibited by NMN at the active site include Asp112, His22, His19, Asp181, Ala184, Ser183, and Ile182. Previous studies of bacterial NNAT have highlighted the crucial role of the conserved residues corresponding to Asp112, His22, and His19 in substrate binding and Asp112 in catalytic function [23,42]. These residues are also observed within the KpNNAT-ATP complex. The largely driven electrostatic interaction observed in the KpNNAT-NMN complex compared to the KpNNAT-ATP complex may indicate slightly tighter binding of NMN to the receptor than ATP. Overall, ATP and NMN exhibit a comparable binding free energy of −20.42 ± 5.05 (kcal/mol) and −20.96 ± 6.18 (kcal/mol), respectively, which shows their binding affinity for KpNNAT receptor are relatively the same. Since the substrates demonstrate an almost equal binding affinity for the KpNNAT receptor, it is possible that the enzyme KpNNAT may also demonstrate a random sequential Bi-Bi mechanism as suggested for Bacillus anthracis NNAT through Kinetic studies [53]. However, further kinetic and direct binding studies will be required to establish this process.

As established from far-UV CD, ANS fluorescence spectroscopy, thermal shift, and activity assays, Ca^2+^ did not alter the conformation of KpNNAT, yet its presence inactivates the enzyme. Zn^2+^ and Cu^2+^ unfold the enzyme as well as suppress its activity. On the other hand, Ni^2+^, which partially unfolds the protein, maintains activity together with Mg^2+^, which did not alter the conformation of the enzyme. In a bid to expound on this and gain further insight into the atomistic impact of these divalent cations on the dynamism of the enzyme and ATP, we employed an MD simulation. We did not have access to parameter files for Cu^2+^ and Ni^2+^ in Maestro when this experiment was carried out. Hence, the MD simulation was performed with only three divalent cations (Mg^2+^, Ca^2+^, and Zn^2+^). Analysis of the apo and the KpNNAT-ATP complex in the absence and presence of the three divalent cations using a 100 ns simulation time showed that all the simulated systems were equilibrated correctly (Table 5), thus suggesting that the extrapolations made from this MD simulation data is reliable.

Based on the Cα RMSD of the simulated systems (Figure 6), the binding of ATP in the presence of the three divalent cations slightly increases the stability of the protein compared to the apo form. This slight stability correlates with the data obtained from the thermal shift assay, which show a 0.5 °C increment in the *T*_m_ of the protein upon ATP binding (Table 3) and the presence of the Mg^2+^ and Ca^2+^. This indicates that ATP binding stabilises the protein’s dynamism, although the impact is minimal. Even though Zn^2+^ denatures the protein as observed in the thermal shift assay (Figure S3), at 100 ns we cannot see the effect of Zn^2+^ in terms of its ability to denature the protein. This is possible because it takes more than 100 ns to be observed which may also account for the minute activity observed in the activity assay. Nevertheless, from this data, we can make a reasonable inference regarding the ligand dynamics, given that the deviation in the RMSD value between KpNNAT apo and KpNNAT-ATP complex in absence and the presence of the metals is within 1–3 Å (Figure S7). It indicates that the protein is still intact within the first 100 ns and did not make any sizeable conformational change greater than 3 Å, which suggest that the protein is stable. There is no significant conformational change in the global structure of the protein.

Furthermore, since the binding of a ligand to protein may either decrease or increase the movement of the molecular structure, the impact of ATP and the divalent cations on the flexibility of the protein structure was evaluated. As revealed from the Cα-RMSF (Figure S8), ATP binding decreases the flexibility of KpNNAT. However, this flexibility reduction differs from the type of divalent cation present. The radius of gyration (Rg) of the protein further lends credence to this, as ATP binding stabilises the protein by preventing it from flapping around, hence increasing its compactness as indicated by the lower Rg in comparison to the apo structure (Figure 7). The presence of divalent cations does not have any noticeable effect on the Rg of KpNNAT apo; however, upon ATP binding, variations were observed in the Rg of the protein, with Zn^2+^ showing a slight decrease in compactness compared to Mg^2+^, Ca^2+^, and no metal. This suggests that ATP binding affects KpNNAT dynamics, and the dynamics of ATP is dependent on the absence or presence of a divalent cation and the type of divalent cation. This is further supported by the ligand RMSD and ligand RMSF data, which showed ATP to exhibit more dynamism in the presence of Mg^2+^ compared to no metal; however, this dynamism diminishes with the replacement of Mg^2+^ with Ca^2+^ and Zn^2+^, hence impacting the activity of the enzyme.

Moreover, details of ATP atoms interactions with the protein residues (Figure 9, Figures S8 and S9) showed that the contacts and interactions made by ATP in the presence of Zn^2+^ and Ca^2+^ enhanced tighter binding of ATP within the receptor, which impacted the dynamism of ATP consequently on the dynamism of the protein. This is further bolstered by the Cα-RMSF (Figure S8) data, which show that the most fluctuated regions were around residues 40–52 for Mg^2+^ and Zn^2+^, and residues in the apo structure 129–145 for Ca^2+^. Upon ATP binding, this region became less fluctuated, while residues 110–120 and 129–138 were observed to fluctuate more in Mg^2+^ but not in Zn^2+^ and Ca^2+^. Interestingly, these KpNNAT residues 40–52, 110–120, and 129–145 correspond to the loop regions 1, 2, and 3, respectively, in *E. coli* and *B. anthracis* NNAT. Ligand-induced conformational changes were observed at these loop regions when comparing the apo structure with the ligand-bound systems, with region 1 forming intimate interactions with the NaMN moiety and region 2 providing interactions with the ATP-ribose.

In contrast, region 3 facilitates the stacking interaction of the corresponding Arg133 (*E. coli*) or Arg134 (*B. anthracis*) with the adenine ring [23,53]. Studies have shown that aside altering the function of a protein through direct interaction with the active site, indirectly disrupting the conformational changes necessary for ligand binding or dissociation, impacts protein functioning. The relatively lower fluctuations observed for Zn^2+^ and Ca^2+^ at residues 110–120 and 129–138 upon ATP binding indicate reduced protein motion at those regions. Hence, restricting the residues’ movement prevents key catalytic residue flexibility that would enhance substrate binding or catalytic function. Thus, impacting on KpNNAT function as demonstrated by the suppressed or inactivity showed in the presence of Zn^2+^ and Ca^2+^. This proposes why Ni^2+^ could still maintain activity despite partially unfolding KpNNAT. The induced conformational changes possibly allow key protein residues to be moved to enhance substrate binding and catalytic function in KpNNAT.

In conclusion, we successfully over-expressed and purified NNAT from *Klebsiella pneumoniae* and explored its structural and functional features alongside the effect of various divalent cations on the activity, dynamics, and ligand-binding of the protein. This study facilitates a better understanding of how divalent cations can impact the mechanism of NNAT, and hence their preferences for specific divalent cations. It also answers the proposition that variation in divalent cation specificity disturbs NNAT structure and contributes to their functioning. We theoretically studied the effect of these divalent cations on the dynamics of KpNNAT using the Schrödinger Desmond molecular dynamics simulation engine. This algorithm implements the parameter files for only the Mg ion, Ca ion and Zn ion (used in this study) as counter ions during the neutralisation step. Having observed that Zn^2+^ and Ni^2+^ produced a similar far-UV CD spectrum regarding the secondary structure content profile, we postulated that any observed trajectory for Zn^2+^ may correlate with Ni^2+^. However, we observed that the 200 ns trajectory time might be too short to follow major shifts or perturbations in the structure of the enzyme, as well as the region that was most affected by these cations. However, we were able to generally show that the dynamics of KpNNAT were slightly altered depending on the divalent cation used in the simulation. The dynamics of ATP within the binding pocket may be influenced by choice of divalent cation in the water box. The data obtained in this study is beneficial for the novel application of the enzyme and as a molecular basis for further evaluation in the design of structure-based inhibitors with therapeutic potential against MDR *Klebsiella pneumoniae*.

## 4. Materials and Methods

### 4.1. Materials

The pET-28a Expression vectors were obtained from Novagen Inc. (Sigma Merck, Johannesburg, South Africa), *E. coli* T7 Express cells obtained from New England Biolab (Inqaba Biotec, Pretoria, South Africa). NMN, ATP, NAD, GTP, alcohol dehydrogenase (ADH), ANS (8-anilino-1-naphthalene sulfonic acid), mant-ATP, and SYPRO Orange were purchased from Sigma Merck (Johannesburg, South Africa). Other biochemicals and reagents used were of analytical grade.

### 4.2. Over-Expression of Recombinant KpNNAT 

The engineered vector used for expressing the recombinant KpNNAT was constructed such that the gene fragment encoding for KpNNAT (Uniprot ID: B5XZR5) was inserted into pET-28a at the *Nco*I and *Bam*HI site, resulting in a sequence with N-terminus 6× His tag and a thrombin cleavage site (Figure S1). Competent *E. coli* T7 cell was transformed with the engineered vector using heat shock. Upon successful transformation, single colonies of transfected *E. coli* T7 cells from the antibiotics selective plates were picked and inoculated into 20 mL 2 × Yeast-Tryptone (2 × YT), supplemented with 30 µg/mL kanamycin. The media was incubated by shaking (37 °C, 250 rpm, 12–16 h). The culture was diluted in a freshly prepared 2 × YT media containing 30 µg/mL kanamycin and incubated (37 °C, 250 rpm) until optical density at 600 nm was 0.6. Over-expression was induced with 0.5 mM isopropyl-β-thiogalactopyranoside (IPTG), and the culture incubated (30 °C, 200 rpm for 6 h). Finally, the cells were harvested by centrifugation (4 °C, 5000× *g*, 10 min). The pellet was resuspended in a 25 mL lysis buffer (Phosphate-buffered saline (PBS), 25 mM imidazole, 0.01% (*w*/*v*) NaN3, pH 7.2) per litre of culture, followed by freezing (−20 °C, 20 h) to facilitate cell lysis. Frozen cells were thawed and then subjected to sonication on ice to lyse the cells. The lysate was centrifuged (4 °C, 18,000× *g*, 15 min) to separate the soluble fraction from the insoluble. Extracts from the lysate, soluble (supernatant) and insoluble (pellet) fractions were analysed on a 12.5% (*w*/*v*) sodium dodecyl sulphate polyacrylamide gel electrophoresis (SDS-PAGE) for expression of the recombinant protein.

### 4.3. Purification of Recombinant KpNNAT

The solubilised cell fraction containing the N-terminus 6× His tagged KpNNAT was subjected to purification by Ni^2+^-IMAC, carried out using low pressure and a gravity pump (Bio-Rad Model EP-1 EconoTM). The Ni^2+^ Sepharose containing resin was pre-equilibrated with the lysis buffer at a flow rate of 4 mL/min. Solubilised cell fraction was loaded onto the column at 2 mL/min flow rate followed by equilibration and washing with 0.1% (*v*/*v*) Tween-20 and 25 mM imidazole at 4 mL/min to remove non-specifically bound proteins. The bound 6× His tagged protein was eluted with 350 mM imidazole at 2 mL/min flow rate, which competitively displaces the 6× His tagged protein bound to Ni^2+^ in the column and then collected in the elution fraction. Eluted fractions containing the recombinant proteins were pooled and dialysed in two changes against 10 mM monobasic sodium phosphate buffer (10 mM NaH_2_PO_4_, 0.01% (w/v) NaN_3_, 1 mM EDTA, pH 7.2) at 4 °C for 3 h and 16 h, respectively, using a 10 kDa molecular weight cut off membrane tubing to remove salts. The first 20 amino acid containing the 6× His tag and the thrombin cleavage sequence was not cleaved from the final KpNNAT in order to maintain the stability of the recombinant enzyme. The purity of the eluted 6× His-KpNNAT was assessed using SDS-PAGE and further quantified spectrophotometrically using the molar extinction coefficient 38055 M^−1^ cm^−1^ obtained theoretically using the ProtParam algorithm [36].

### 4.4. Enzyme Assay, Optimal pH, and Effect of Divalent Metal Ions

Recombinant KpNNAT was assayed for NNAT activity (synthesis of NAD^+^) using a dual enzyme catalysed reaction coupled to alcohol dehydrogenase (ADH), which enables the quantification of activity based on NADH production. Given that most NNATs can utilise either NaMN or NMN substrate to synthesise NAD^+^, NMN, and ATP were the substrates explored in this study for two major reasons: (i) NMN will require only ADH in this assay system because NaMN will be converted to NaAD^+^, which will be converted to NAD^+^ before NAD^+^ is subsequently converted to NADH and (ii) NaNM is very expensive in comparison to NMN.

A standard assay was carried out in a 1 mL reaction volume containing 0.1 M NaH_2_PO_4_, 0.01% (*w*/*v*) NaN_3_, 5 mM MgCl_2_, 1 mM ATP, 1% (*v*/*v*) ethanol, 10 U/mL ADH, and 1 mM DTT, with a pH of 8.0. The concentration of purified protein used was varied from 0–27 µg/mL and reactions were initiated by the addition of 0.5 mM NMN at 20 °C. An increase in NADH synthesis from the reduction of NAD^+^ produced was monitored at 340 nm using a spectrophotometer for 60 sec at an interval of 0.5 sec.

To determine the activity of the enzyme with respect to pH, a 1 mL standard assay was performed in 0.1 M sodium acetate buffer (pH 5.5), 0.1 M sodium phosphate buffer (pH 6.0–8.0), 0.1 M Tris-HCl buffer (pH 8.5–9.0), and 0.1 M sodium glycine buffer (pH 9.0–10.5) using 27 µg/mL of the recombinant protein. 

The effect of divalent cations on the activity of KpNNAT between pH 5.0 and 9.0 was evaluated by preparing each 2 mM metal salts (CaCl_2_, MgCl_2_, CuSO_4_, NiSO_4_, and ZnSO_4_) in 50 mM sodium acetate (pH 5.0–6.0), and 50 mM Tris-HCl (pH 7.0–9.0) using 27 µg/mL of the recombinant protein. All assays were carried out in triplicates and averaged. The negative control was carried out in the absence of metal. Furthermore, given that ADH is maximally active at pH 9.0, its activity was assessed in the absence and presence of the various divalent metals in 50 mM Tris-HCl, pH 9.0, as a control.

### 4.5. Circular Dichroism Spectroscopy

To analyses the secondary structure content of the recombinant purified KpNNAT, 5 µM dialysed fraction of the protein was prepared in ddH_2_O and subjected to far-UV CD spectroscopy using CD spectropolarimeter (20 °C, 5 nm bandwidth, 1 nm data pitch, 100 nm/min scanning speed). CD spectra were collected between 180 and 255 nm using a 1 mm path length high transparency CD quartz cuvettes under continuous nitrogen flush to remove oxygen. To determine the effect of selected divalent cations on the structure of the protein, 5 mM of salt (MgCl_2_, or CaCl_2_, or NiSO_4_, ZnCl_2_, or CuSO_4_) of pH 7.5 was added to the sample. The conformation of the denatured proteins was also examined by treating protein samples with 8 M urea under the same conditions. All assays were carried out in triplicates and averaged. The resulting spectra data were deconvoluted using the Dichroweb analysis program, CONTIN-LL, to derive the fraction of amino acid involved in the formation of α-helices, β-strands, and loops. Collected data were corrected by subtracting the baseline corresponding to the buffer from the data. The resulting CD spectra were processed using SigmaPlot 12.0 and the final plot generated via GraphPad Prism v8.4.3.

### 4.6. Fluorescence Spectroscopy—ANS and Mant-ATP Binding Studies

The hydrophobicity, structural conformation, and interaction of KpNNAT with ligand molecules were probed by fluorescence spectroscopy using ANS and mant-ATP fluorescent dye. Fluorescence spectra were measured with 10 mm path length quartz cuvettes using a spectrofluorometer (20 °C, 200 nm/min scan speed, 2.5 nm excitation and emission bandwidths, and 1 nm data pitch). ANS binding studies was performed in the absence and presence of ATP, NMN, as well as divalent cations including Mg^2+^, Ni^2+^, Ca^2+^, Cu^2+^, and Zn^2+^ in 25 mM Tris-HCl, 0.01% NaN_3_, pH 7.5. Each sample contained 50 µM ANS and 5 µM dialyzed protein supplemented with either 8 M urea, 1 mM ATP, 1 mM NMN, or 2 mM of salt (MgCl_2_, NiSO_4_, CaCl_2_, ZnSO_4_, and CuSO4). To all the samples containing nucleotide ligand, 2 mM MgCl_2_ was added. Blanks were prepared for the respective samples by excluding 5 µM protein. Upon excitation of the sample at 395 nm, ANS fluorescence spectra were obtained between 400 and 650 nm emission wavelength. 

The binding of mant-ATP to KpNNAT was studied in the absence and presence of ATP. The prepared samples contained 10 µM mant-ATP and 2.5 µM protein analysed in 10 mM NaH_2_PO_4_, 0.01% NaN_3_, pH 7.5. This reaction mixture was supplemented with 8 M urea and 1 mM of ATP. To the sample containing ATP, 5 mM MgCl_2_ was added. All samples were excited at 355 nm with emission spectra collected between 400 and 600 nm. 

In all binding studies, three emission spectra were collected average and corrected for the corresponding free ANS/mant-ATP and buffer absorption. The resulting spectra were processed using SigmaPlot and the final plots generated via GraphPad Prism v8.4.3. The maximal peak of the fluorescence emission and the change in quantum yield were used in monitoring the folding processes of the enzyme.

### 4.7. Thermal Shift Assay

Thermal shift assay was carried out using the BioRad CFX96 Touch Real-Time PCR Detections System (Bio-Rad Laboratories, Inc., Hercules, CA, USA), and the data were subsequently analysed using the BioRad CFX-Maestro software. All assay experiments used 20 µg of recombinant KpNNAT (with exception of the no protein blanks) solubilised in 25 mM Tris HCl, pH 7.5 and 0.02% (*w*/*v*) sodium azide. The following divalent metal salts were added to a final concentration of 5 mM; MgCl_2_, or CaCl_2_, or NiSO_4_, ZnCl_2_, or CuSO_4_. In addition, ATP (0.1 mM) was supplemented in the buffer and 5 µL of 50× Sypro Orange (Sigma) up to a total volume of 25 µL. All assays were carried out in four replicates. The PCR plates were sealed with optical seal, gently vortexed for 5 s, and centrifuged. Thermal scanning (20 °C to 80 °C at 0.5 °C for 10 s increments) was performed using a real-time PCR setup (CFX384, BioRad) and fluorescence intensity was measured after every 10 s. Curve fitting, melting temperature calculation and report generation on the raw relative fluorescence unit (RFU) and the –d(RFU)/dT data were performed using BioRad CFX-Maestro software. The plots were created using GraphPad Prism v8.4.3.

### 4.8. Molecular Modelling Studies of KpNNAT 

The computational work was executed using high end desktop servers (16 CPU Intel^®^ core i7™, 3.3 GHz, 20 M cache), MSI X99 motherboard with 32 GB DDR4-2133 MHz memory and 264 GB RAM, Nvidia GTX 750Ti graphics card, Schrödinger Maestro v12.2, and Linux server (2019 GPU-enabled Schrödinger Desmond) (Council for Scientific and Industrial Research, Cape Town, South Africa).

#### Homology Modelling of KpNNAT, Protein and Ligand Preparation

The fasta sequence of KpNNAT was extracted from Uniprot.org (assess number: A6T6A0) ID 6RWD) and submitted to the Swiss Model homology modelling engine (REF) for homology modelling. The top-scoring template in the protein data bank, 1K4K, corresponding to the *E. coli* homologue with over 76% sequence identity, was used to develop a homology model for KpNNAT. The KpNNAT model was submitted to ProCheck [54] for stereochemical analysis using the Ramachandran analysis. The model was subsequently submitted to the protein preparation wizard module implemented in Maestro v12.2 molecular modelling algorithm. The protein structure was pre-processed by assigning bond orders, adding hydrogen atoms, creating zero bond orders to metals and disulfide bonds, and deleting water molecules that were 5 Å from heterogen atoms. Further, the hydrogen bonding network was optimised by sampling the orientation of water molecules using the PROPKA algorithm at pH 7.0. Finally, the structure was refined by minimisation using the OPLS_2005 force field, such that restrains were placed on the heavy atoms. The heavy atoms could converge using a RMSD of 0.3 Å cut-off for terminating the minimisation process. The stereochemistry of the side chains was checked to ensure that no major perturbations have been induced while preparing the structure. The minimised structure was saved as Maestro (.mae) file for subsequent prediction analysis.

For ligand preparation, the structure data files (SDF) corresponding to ATP(CID:5957) and β-nicotinamide mononucleotide (CID:14180) were extracted from the PubChem database and submitted to the LigPrep module implemented in Maestro v12.2 for ligand preparation, which involves energy minimisation using the OPLS_2005 force field. The algorithm was set to generate possible states of each ligand at pH 7.0 ± 2, while accurately predicting the pKa of these states at the set pH using the Epik module of the algorithm. The ligands were also desalted, and possible tautomeric states (~3 tautomers/ligand) were further generated at pH 7.0 ± 2. Additionally, specific chiral centres were retained (for molecules with multiple chiral centres), while other chiral centres were varied during the ligand preparation to return chemically sensible structures with low energy states. These generated molecules were saved as individual Maestro (.mae) files for induced fit ligand docking.

### 4.9. Induced Fit Ligand Docking

Induced fit ligand docking (as implemented in Schrödinger Maestro v12.2 algorithm) was used to predict the binding of ATP and β-nicotinamide mononucleotide (NMN) to KpNNAT. This is because the landscape geometry of the ligand-binding site in a protein critically depends upon conformational changes that the ligands induce in the protein structure upon binding. An implicit solvent model using the OPLS_2005 force field was used for this process. The induced-fit, ligand-docking protocol applied ring conformational sampling with a 2.5 kcal/mol energy barrier and a non-planar conformation penalty on amide bonds. The scaling for both receptor and ligand was set at 0.5 with a maximum of 20 allowable poses per ligand. Residues within 5.0 Å of the docked ligand were further refined using the Prime Refinement algorithm (implemented in Maestro v12.2). Prime energy algorithm (implemented in Maestro v12.2) was additionally applied to rank the refined protein-ligand complexes. The receptor structure within 30.0 kcal/mol of the minimum energy structure were submitted for a final round of Glide docking and scoring. Each ligand was re-docked into every refined low-energy receptor structure in the subsequent second docking step using the default Glide XP settings.

### 4.10. Molecular Dynamic Simulation

Molecular dynamic (MD) simulations were carried out using the GPU-enabled, Desmond molecular dynamics simulation engine implemented in Maestro v12.2. The complex corresponding to the top-scoring pose for KpNNAT:ATP or KpNNAT:NMN or KpNNAT- apo was saved as .mae files and submitted to a Linux (Ubuntu) desktop server for the Desmond MD simulations studies. Prior to the MD simulation, each of the three systems for simulation (KpNNAT:ATP, KpNNAT:NMN, or KpNNAT-apo) were built using the System Builder module implemented in the Desmond algorithm. This solvated the system using the TIP3P-explicit solvent model using the OPLS_2005 force field. The KpNNAT -ligand complex or KpNNAT-apo was placed in an orthorhombic box (distance from the box face to the outermost protein/ligand atom was set 10 Å, the box angle was α = β = γ = 90°). The volume box containing the KpNNAT -ligand complex or apo- KpNNAT was minimised, and counter ions (placed at least 20 Å from each ligand) added to neutralise the system. The system was physiologically conditioned by adding 0.15 M NaCl or MgCl_2_ or ZnCl_2_ or CaCl_2_ into the solvent box. After the solvation and ionisation phase in the explicit solvent model, the system was submitted for the molecular dynamics production phase. The MD simulation phase is divided into eight distinct stages in which the simulation parameters were specified for each stage. Stages 1–7 are the equilibration, which comprises short simulation steps, and stage 8 is the final long range 100 ns simulation stage. The type and parameters of the solvated system were detected in stage 1. In stage 2, a 100 ps simulation was carried out using Brownian Dynamics under NVT condition at 10 K with restraints placed on the solute heavy atoms. Stage 3 involved a 12 ps simulation under NVT condition at 10 K with restraints on heavy atoms. Stages 4, 6 and 7 (the pocket solvation at stage 5 was skipped) employed short simulation steps (12, 12 and 24 ps, respectively) under NPT conditions (at 10 K and restraints on heavy atoms for stages 4 and 6). No restraints were placed on heavy atoms at stage 7. The final MD production stage at constant temperature of 300 K was carried out at stage 8.

### 4.11. Post Dynamic Analysis

Post dynamic analyses of the trajectories derived from the MD simulation studies were performed with Schrodinger Maestro v12.2. Essentially, (i) the quality of the simulation, which analyses the average energy, pressure, temperature and volume of each simulated system were analysed using Simulation Quality Analysis (implemented in Maestro v12.2). (ii) The root-mean-square-deviation (RMSD) of the alpha carbon atoms (Cα), RMSD of the ligand with respect to the receptor, the root-mean-square fluctuations (RMSF) of the residues, secondary-structure element analysis and protein-ligand interaction analysis were performed using the Simulation Interaction Diagram algorithm (implemented in Maestro v12.2). The radius of gyration (Rg) and atomic distance calculations were performed using the Simulation Events Analysis algorithm (also implemented in Maestro v12.2). The average of a representative cluster was determined using the Cα RMSD trajectory clustering algorithm implemented in the Desmond.

### 4.12. MM/GBSA Binding Free Energy Calculations

The molecular mechanics/generalised Born solvent area (MM/GBSA) method implemented in Amber 18 was used to calculate the binding free energy (Δ*G*_bind_) to gain more insight into the ligands’ binding KpNNAT. Briefly, the free energy of the binding of ATP and NMN to KpNNAT were calculated by averaging 2000 snapshots from the 20 ns trajectory of the simulated complexes. The ΔG_bind_ of ligands at the KpNNAT active site was calculated by using (1)

∆*G*_bind_ = Δ*G*_RL_ − (Δ*G*_R_ + Δ*G*_L_) (1)

where Δ*G*_RL_, Δ*G*_R_, and Δ*G*_L_ represent the free energies of the complex, receptor, and ligand, respectively. The free energy (*G*) of each state was derived using Equations (2)–(5):

∆*G*_bind_ = ∆*E*_gas_ + Δ*G*_sol_ − *T*Δ*S* (2)

*E*_gas_ = *E*_int_ + *E*_vdW_ + *E*_ele_ (3)

*G*_sol_ = *G*_GB_ + *G*_SA_ (4)

*G*_SA_ = γSASA (5)

The ff14sb force field terms were used to estimate the gas-phase energy (Δ*E*_gas_), which was the sum of the internal energy (Δ*E*_int_) the Coulomb energy (Δ*E*_ele_), and the van der Waals energies (Δ*E*_vdW_). The energy contribution from the polar states (*G*_GB_) and non-polar states (*G*_SA_) was employed to evaluate the solvation free energy (Δ*G*_sol_). The non-polar term was estimated from a linear relation to the solvent accessible surface area (SASA in Å2), which was used to derive the non-polar solvation energy (*G*_SA_) using a water probe radius of 1.4 Å. The contribution from polar solvation (*G*_GB_) was determined by solving the generalised Born equation, where the total entropy of the solute and temperature was represented by *S* and *T*, respectively. Per residue-free energy decomposition was carried out using the MM/GBSA method (for binding residues) in Amber 18 to obtain the contribution of each residue at the dimer interface to the total binding free energy profile between the ligands and KpNNAT.

## Figures and Tables

**Table 1:** Influence of divalent cations on the activity of KpNNAT at different pH.

Divalent Cation	Concentration (mM)	^1^ Relative Activity (%)				
		pH 5.0	pH 6.0	pH 7.0	pH 8.0	pH 9.0
Mg^2+^	2	100	100	100	100	100
None	0	0	0	0	0	0
Ni^2+^	2	0	300	61	182	47
Ca^2+^	2	0	0	0	4	0
Zn^2+^	2	67	250	4	27	2
Cu^2+^	2	333	50	33	1	1

^1^ Relative activity was assayed in the presence of 1 mM ATP and 0.5 mM NMN at 20 °C. The analysis was recorded with respect to Mg^2+^ (100%).

**Table 2:** Influence of divalent cations on the secondary structure of KpNNAT.

Secondary Structure Element	Secondary Structure Content (%)					
	No Divalent Cation	Mg^2+^	Ni^2+^	Ca^2+^	Zn^2+^	Cu^2+^
Helix	36.3	31.7	18.1	36.8	17.3	4.1
Sheets	16.9	14.5	21.1	17.4	22.3	34.7
Turns	24.4	20.4	16.3	21.4	16.2	15.6
Unordered	22.5	33.4	44.5	24.5	44.2	45.6

**Table 3:** Showing the melting temperature (*T*_m_) for KpNNAT in the absence and presence of ATP and varying divalent cations metal.

Divalent Cation	*T*_m_ (°C)	
	Apo	ATP
None	40.0	40.5
Mg^2+^	40.5	40.5
Ca^2+^	41.0	40.5
Ni^2+^	39.0	39.5
Zn^2+^	ND	ND
Cu^2+^	ND	ND

**Table 4:** Ramachandra plot summary for the KpNNAT model.

Regions on the Ramachandran Plot	Number of Residues (%)
Most favoured	168 (91.80)
Additional allowed	14 (7.70)
Generously allowed	1 (0.50)
Disallowed regions	0 (0.00)

**Table 5:** The simulation quality analysis of KpNNAT-apo and KpNNAT-ATP complex in the absence and presence of divalent cations. This table shows the mean, standard deviation (SD), and slopes of total energy (Et), Potential energy (Ep), temperature (T), and volume (V) of the systems over 100 ns simulation time.

Divalent Cation	Parameter	Apo		ATP	
		Mean (±SD)	Slope (ps^−1^)	Mean (±SD)	Slope (ps^−1^)
None	Et (kcal/mol)	−70,875.96 (103.64)	−0.001	−70,901.56 (102.27)	0.000
	Ep (kcal/mol)	−87,147.54 (79.33)	−0.001	−87,199.87 (78.19)	0.000
	T (K)	298.70 (0.83)	−0.001	298.70 (0.82)	0.000
	V (Å)	264,588.91 (321.42)	−0.001	264,640.84 (331.21)	0.002
Mg^2+^	Et (kcal/mol)	−80,676.69 (102.83)	−0.001	−80,706.63 (104.13)	−0.001
	Ep (kcal/mol)	−96,937.15 (79.17)	−0.001	−96,996.64 (90.94)	−0.001
	T (K)	298.70 (0.82)	0.000	298.69 (0.82)	0.000
	V (Å)	263,554.46 (319.05)	0.001	263,919.64 (328.31)	−0.001
Ca^2+^	Et (kcal/mol)	−78,760.34 (102.22)	0.000	−78,811.46 (103.08)	0.000
	Ep (kcal/mol)	−94,975.14 (78.41)	0.000	−95,055.73 (79.09)	0.000
	T (K)	298.71 (0.82)	0.000	298.71 (0.83)	0.000
	V (Å)	263,178.04 (363.80)	0.005	263,467.74 (345.74)	0.005
Zn^2+^	Et (kcal/mol)	−80,233.29 (103.89)	0.000	−80,281.10 (103.85)	0.000
	Ep (kcal/mol)	−96,491.55 (79.85)	0.000	−96,569.21 (79.81)	0.000
	T (K)	298.70 (0.82)	0.000	298.69 (0.83)	0.000
	V (Å)	26,367.74 (323.55)	0.000	263,992.61 (330.07)	−0.001

**Table 6:** The binding free energy profile (bold) of KpNNAT complex with ATP and NMN, as calculated using MM/GBSA method 7.

Systems	Energy (kcal/mol)				
	∆*E*_vdw_	∆*E*_ele_	∆*E*_gas_	∆*E*_sol_	∆*E*_bind_
KpNNAT:ATP	−34.61 ± 3.62	−59.11 ± 7.89	−93.72 ± 7.13	73.30 ± 5.19	**−20.42 ± 5.05**
KpNNAT:NMN	−20.86 ± 3.32	−103.96 ± 21.27	−124.80 ± 20.87	103.85 ± 16.70	**−20.96 ± 6.18**

## Data Availability

Not applicable.

## Supplementary Materials

The following are available online at https://www.mdpi.com/article/10.3390/ijms23010116/s1.
