# Supplementary material for: Effect of Divalent Metal Ion on the Structure, Stability and Function of Klebsiella pneumoniae Nicotinate-Nucleotide Adenylyltransferase: Empirical and Computational Studies
Source: Int J Mol Sci. 2021 Dec 23;23(1):116. doi: 10.3390/ijms23010116 (PMC8745210; doi:10.3390/ijms23010116)
Supplement: Supplementary file 1 [file ijms-23-00116-s001.zip › ijms-1450243 - supplementary.pdf]

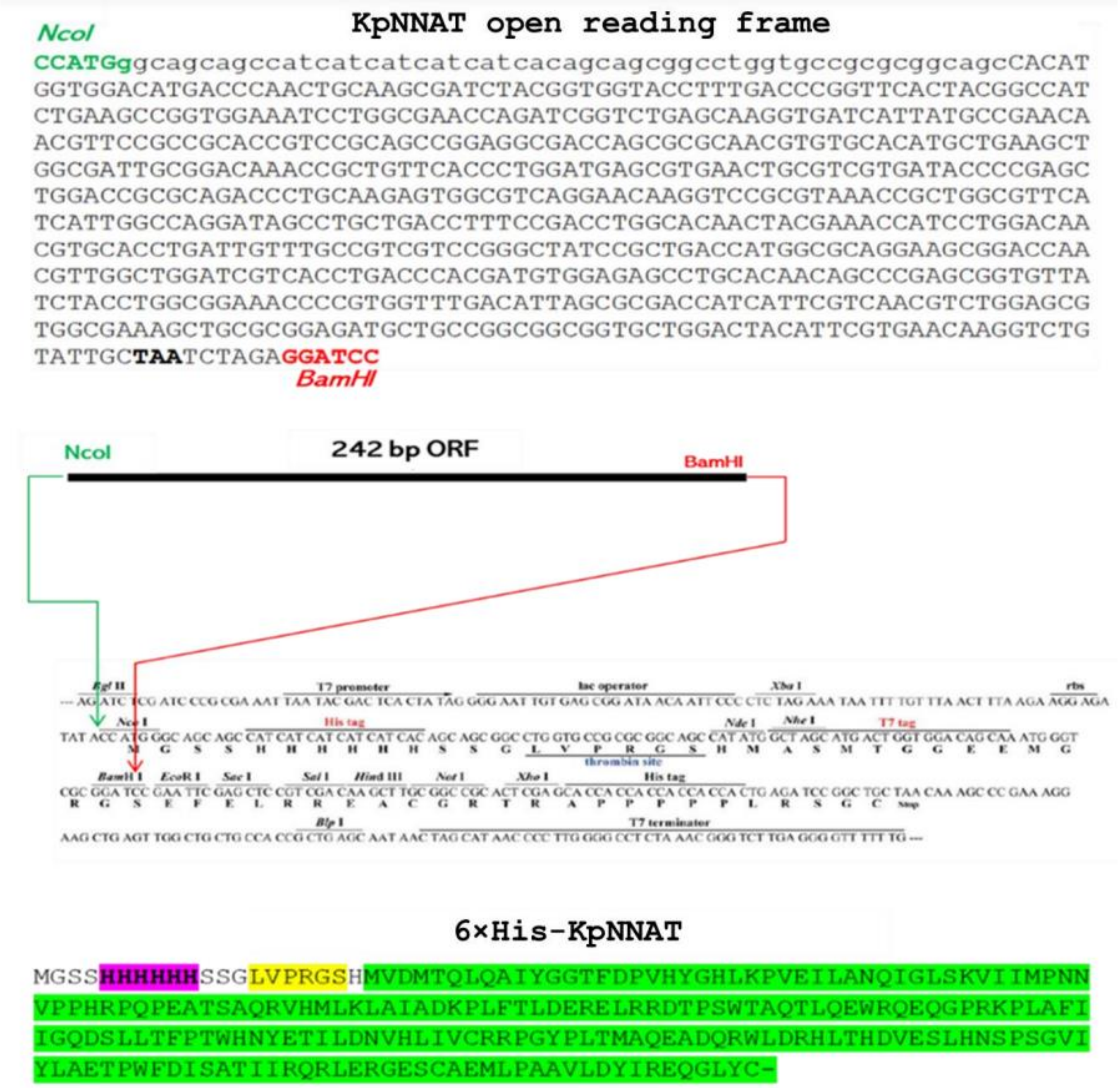

Figure S1. Picture depicting the constructed vector used for expressing recombinant KpNNAT. The gene fragment encoding for KpNNAT was inserted into pET-28a at the NcoI and BamHI sites resulting in a KpNNAT sequence with a hexahistidine as indicated in purple and a thrombin cleavage site indicated in yellow.

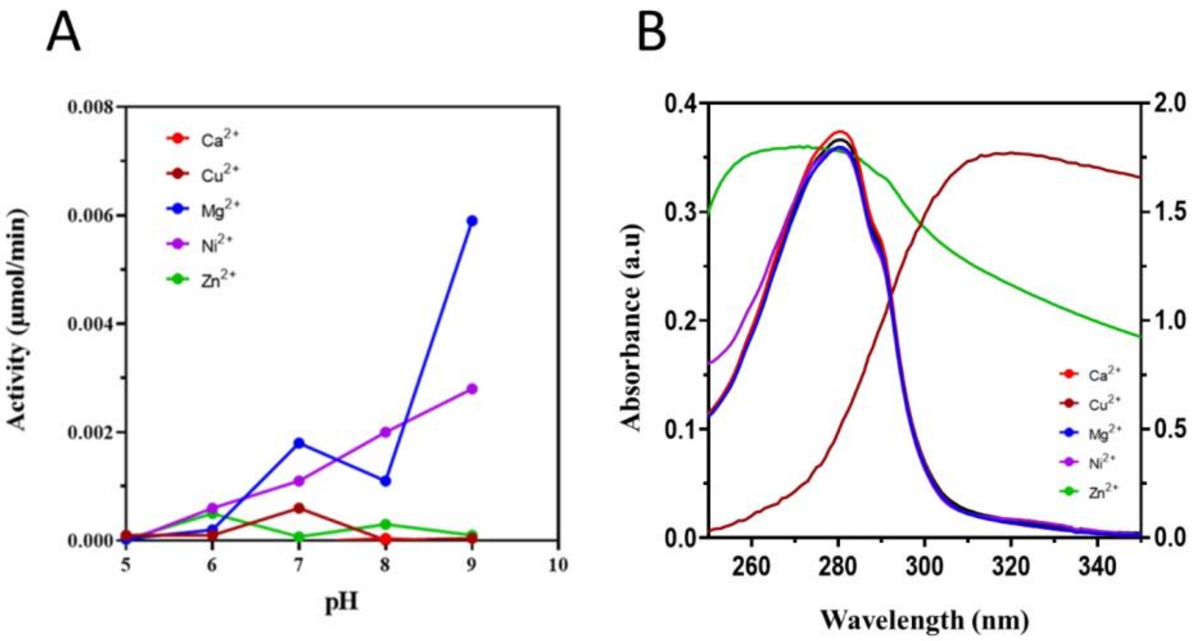

Figure S2. (**A**) The activity profile of KpNNAT in the presence of different divalent metal ions at varying pH. Analysis was carried out spectrophotometrically using ATP and NMN as substrates in the presence of 2 mM Ca^{2+}, Cu^{2+}, Mg^{2+}, Ni^{2+}, and Zn^{2+}. (**B**) The UV-visible spectra of 250 μM KpNNAT in the presence of 5 mM Ca^{2+}, Cu^{2+}, Mg^{2+}, Ni^{2+}, and Zn in 50 mM Tris-HCl, pH 7.5. The plots were generated using GraphPad Prism.

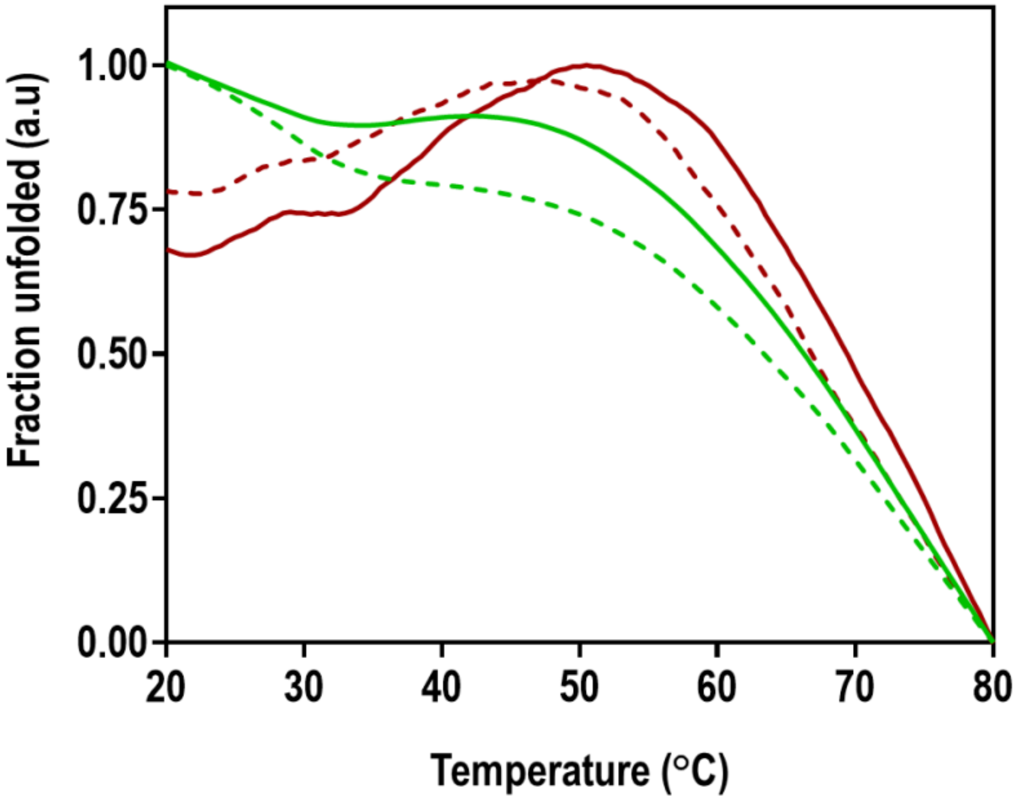

Figure S3. Thermal shift assay of KpNNAT showing the unfolding transition of the protein in the presence of Zn^{2+} (green) and Cu^{2+} (brown). The lack of a sigmoidal curve and a high initial RFU values shows that the protein is denatured at room temperature. The dotted lines indicate absence of ATP while the solid lines indicate presence of ATP.

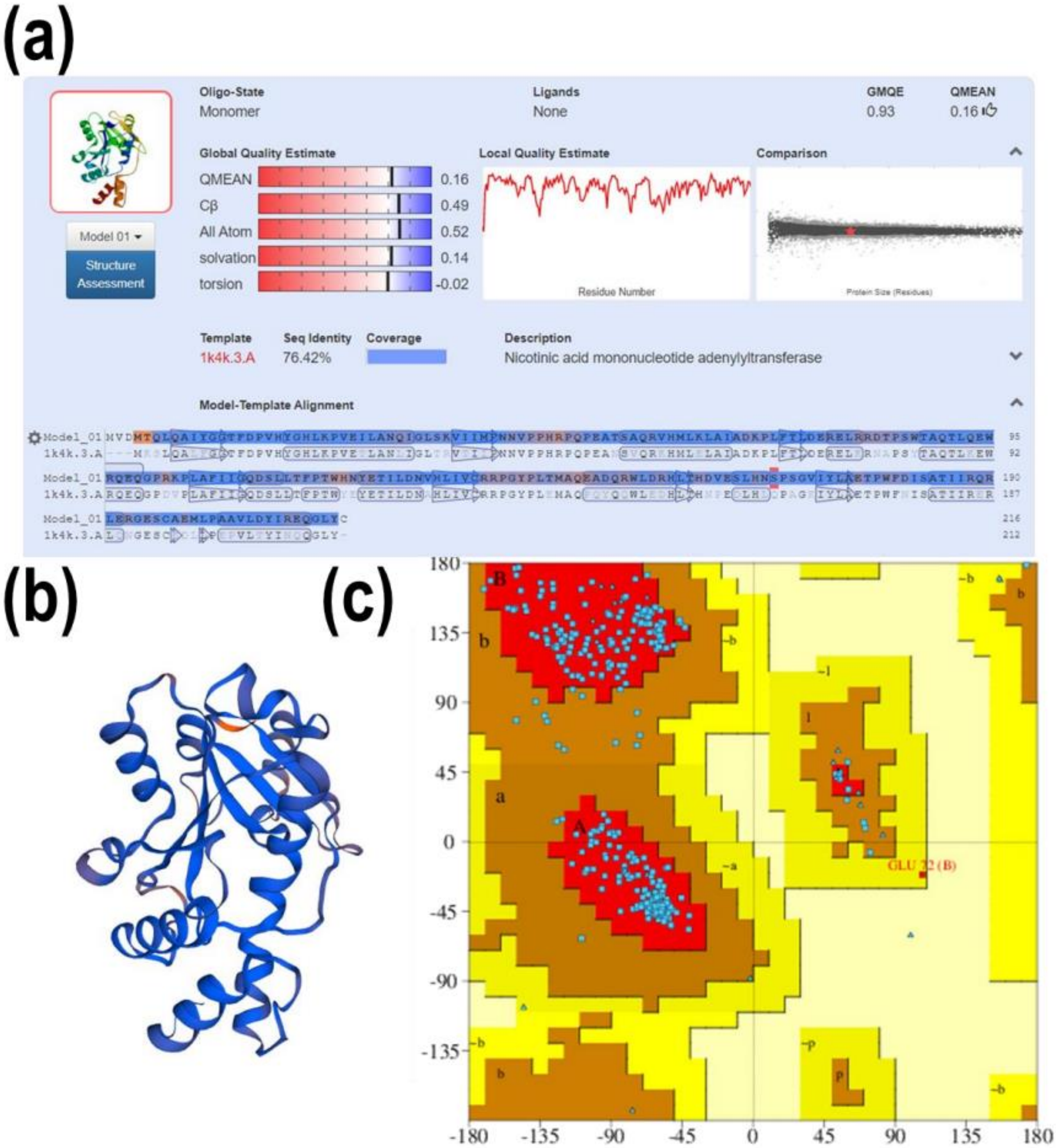

Figure S4. Validation of KpNNAT model through (**a**) Swiss modelling which indicate a high sequence identity of 76.42%, a QMEAN of 0.16 and GMQE of 0.93 between the modelled structure (KpNNAT) and the template used (EcNNAT) (**b**) Structural alignment with the template which showed a RMSD of 0.082 Å over 201 residues when superimposed on each via PyMol and (**c**) Ramachandran plot statistics which analyses the stereochemistry and geometry of the side chains with a data showing more than 90% of the residues in the most favour region, and no residue in the disallowed regions.

(a)

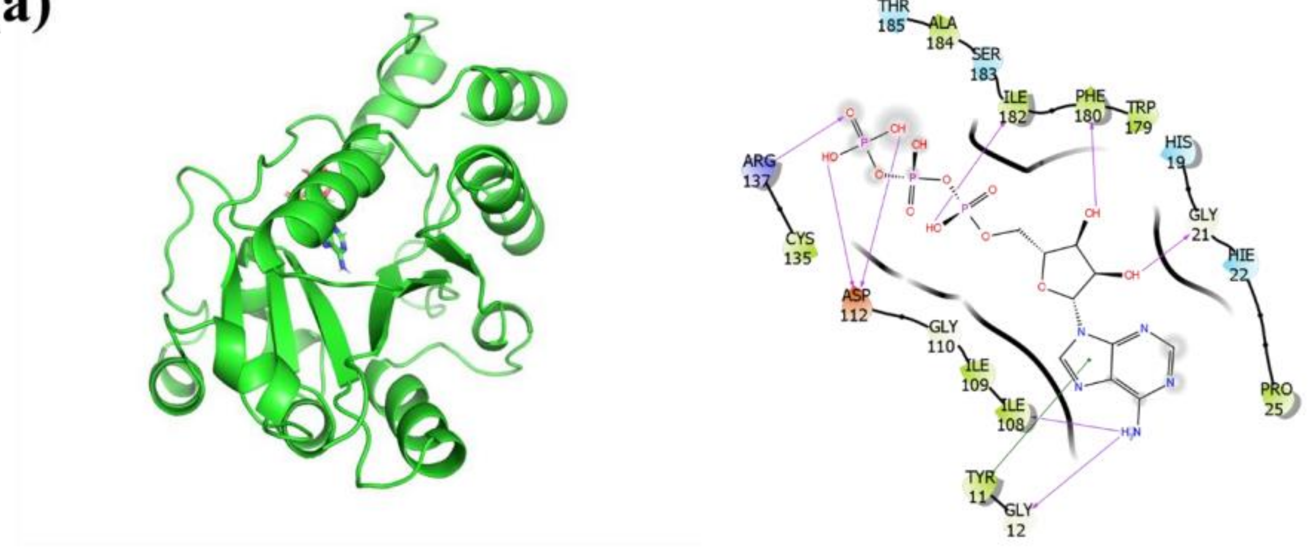

(b)

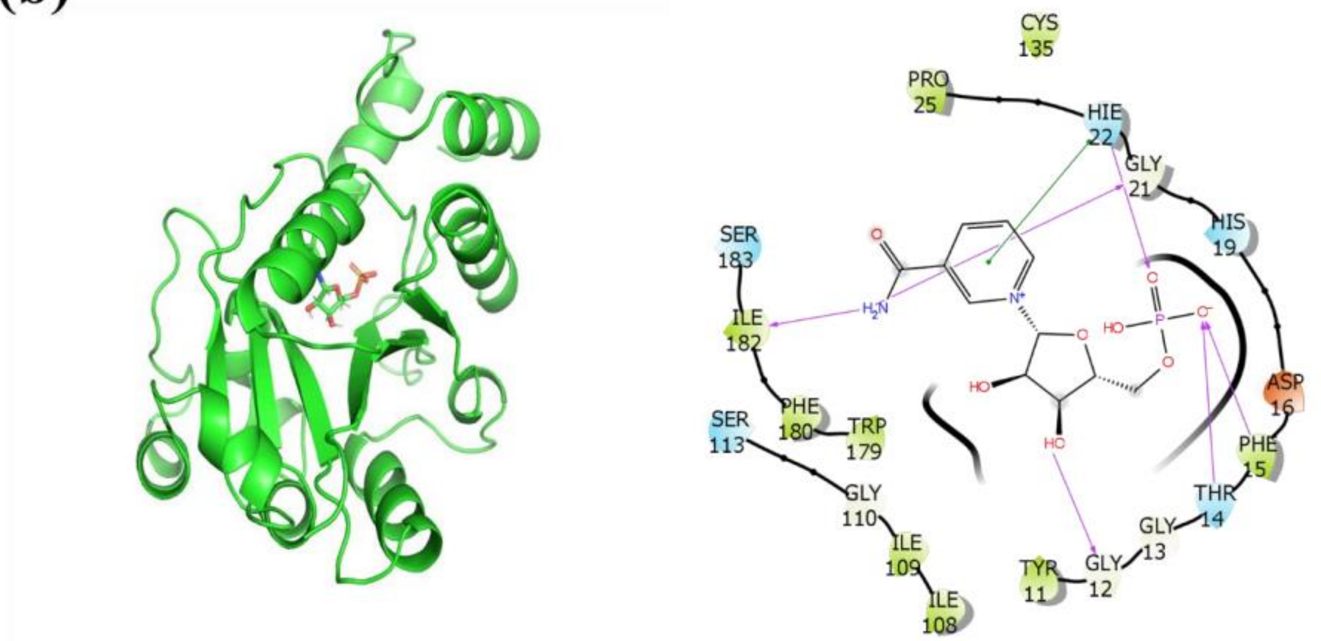

Figure S5. Ribbon representation versus 2-dimensional interaction plot of the induced-fit docking of (**a**) KpNNAT-ATP and (**b**) KpNNAT-NMN complexes. The ligands are shown in stick representation. The amino acid residues are within 4 Å from the ligand and are represented as follows: blue is polar, green is non-polar, orange is negatively charged, and violet is positively charged. The hydrogen bond is represented as purple lines, salt bridge interactions as blue-red lines, green lines represent π- $\pi*$ interaction, while exposure to solvent is indicated in gray. The ribbon representation was generated using PyMol, while the 2-D images were produced from the Maestro 2D interaction diagram.

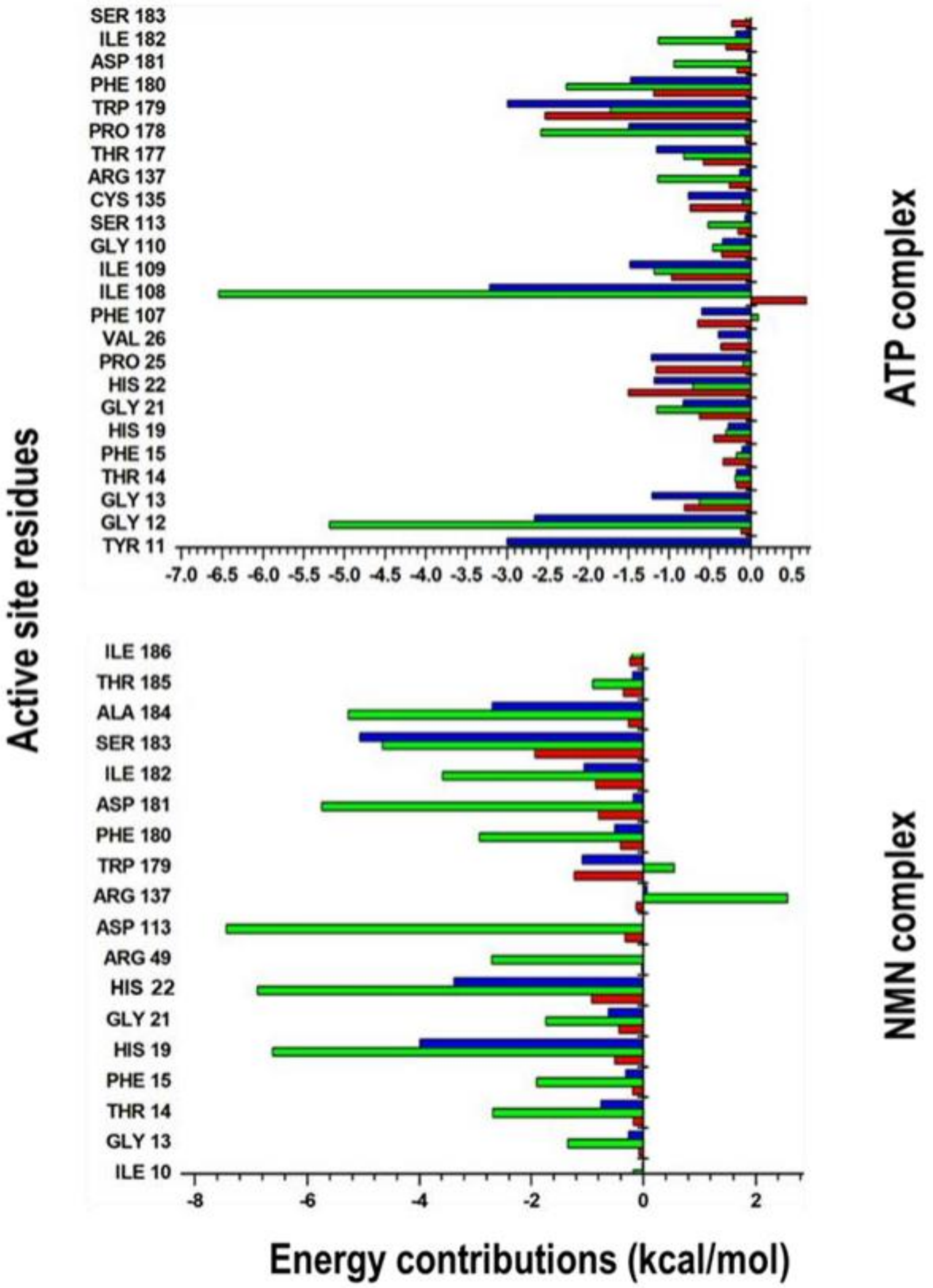

Figure S6. Graphical representation of the total energy contribution of KpNNAT binding site residues interacting with ATP and NMN as derived using the MM/GBSA method in Amber 18. The green bars indicate electrostatic interactions, the red bars indicate van der Waals interactions, and the blue bars indicate the total energy. The plots were extracted from 2000 snap shots of a 20 ns MD simulation.

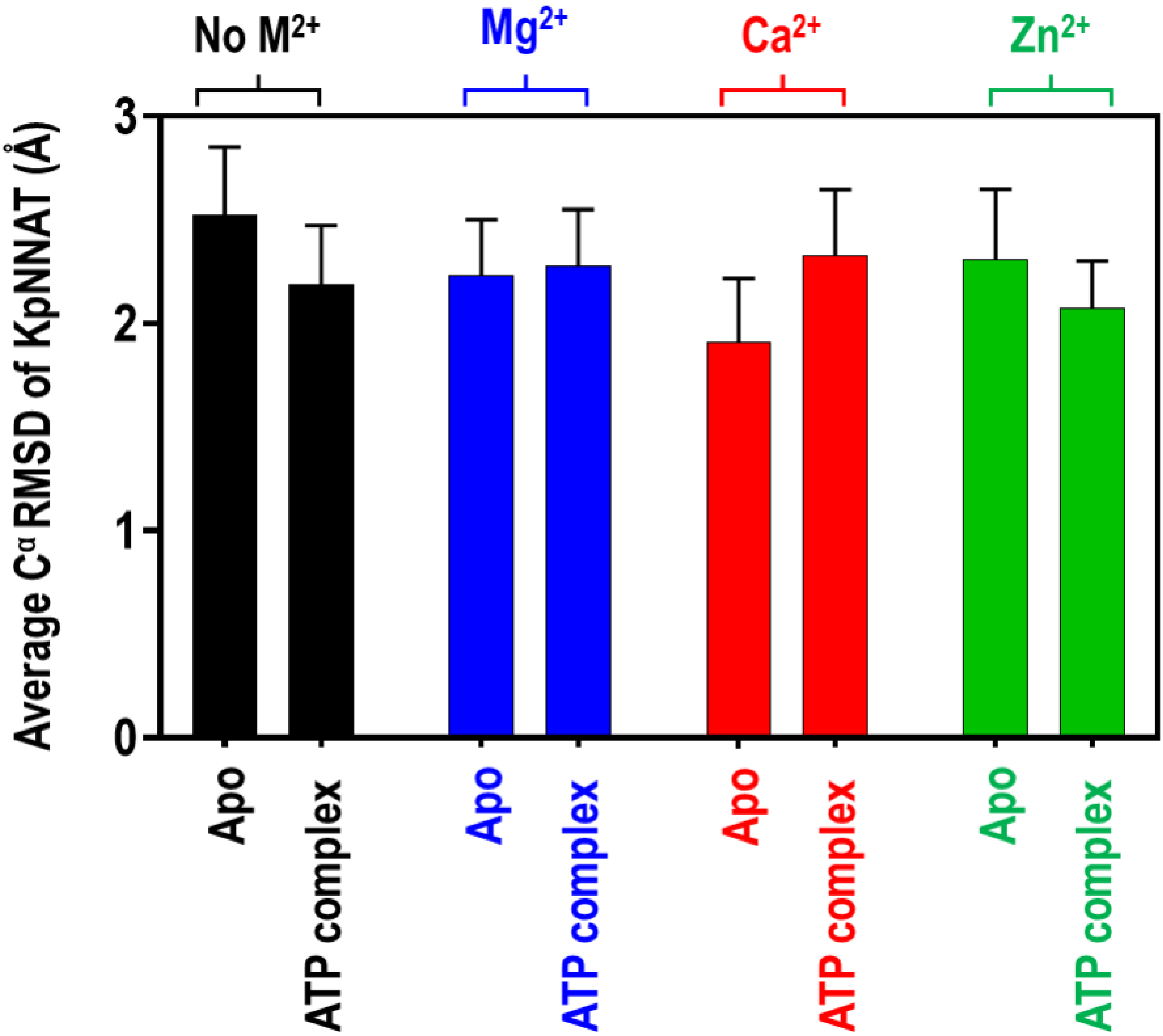

Figure S7. Bar chart of the average Cα RMSD of KpNNAT apo and KpNNAT-ATP complex over a duration of 100 ns MD simulation in the absence of metal ion (black) and presence of Mg^{2+} (blue), Ca^{2+} (red), and Zn^{2+} (green).

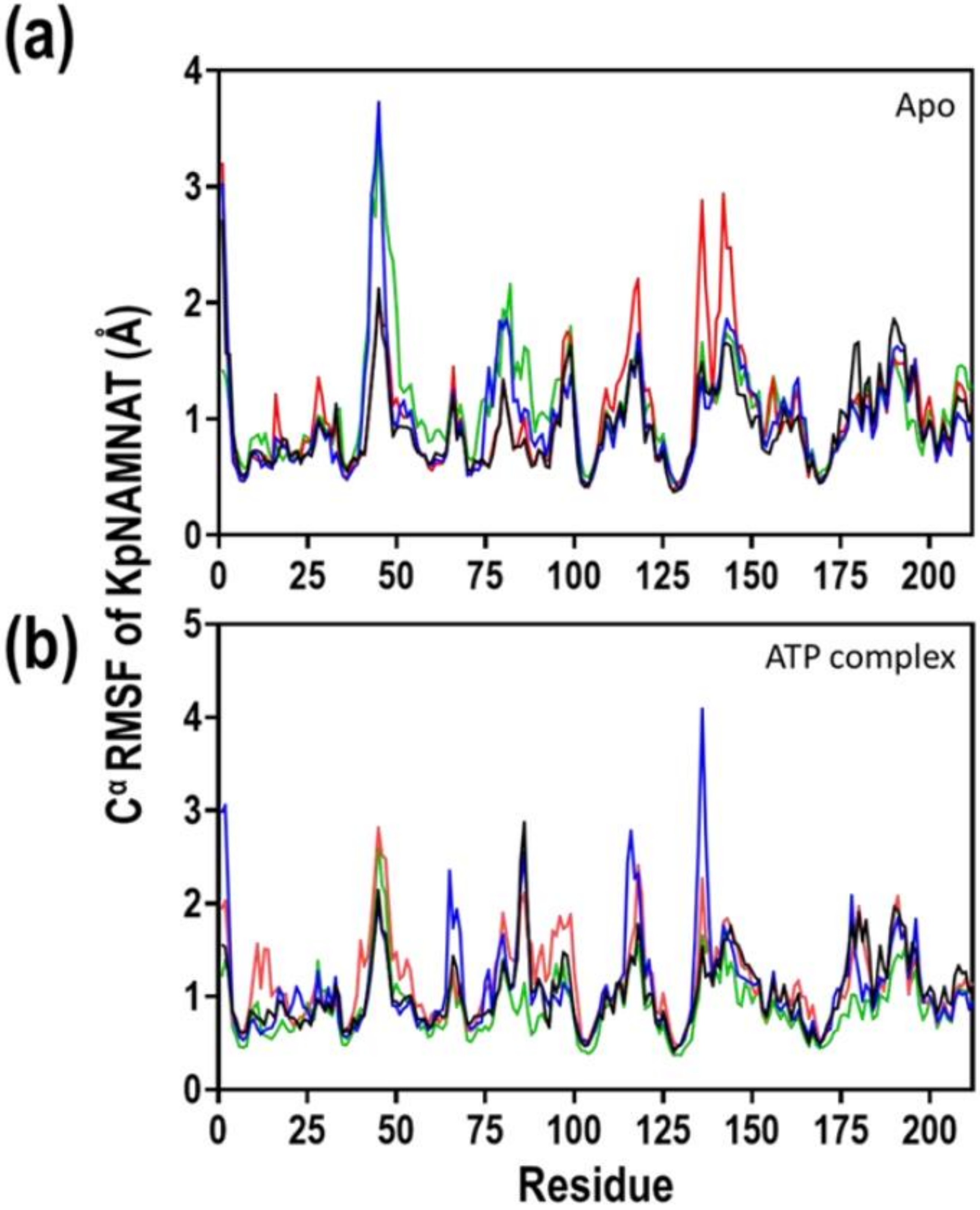

Figure S8. The root-mean-square fluctuation (RMSF) of the Cα atoms of KpNNAT over 100 ns simulation time. Analysis of KpNNAT apo and KpNNAT-ATP complex in the absence (black) and presence of Mg^{2+} (blue), Ca^{2+} (red), and Zn^{2+} (green) showed fluctuations of the Cα atoms within regions 112–120 and 129–135 residues upon ATP binding in the presence of the metals. The plots were generated using GraphPad Prism.

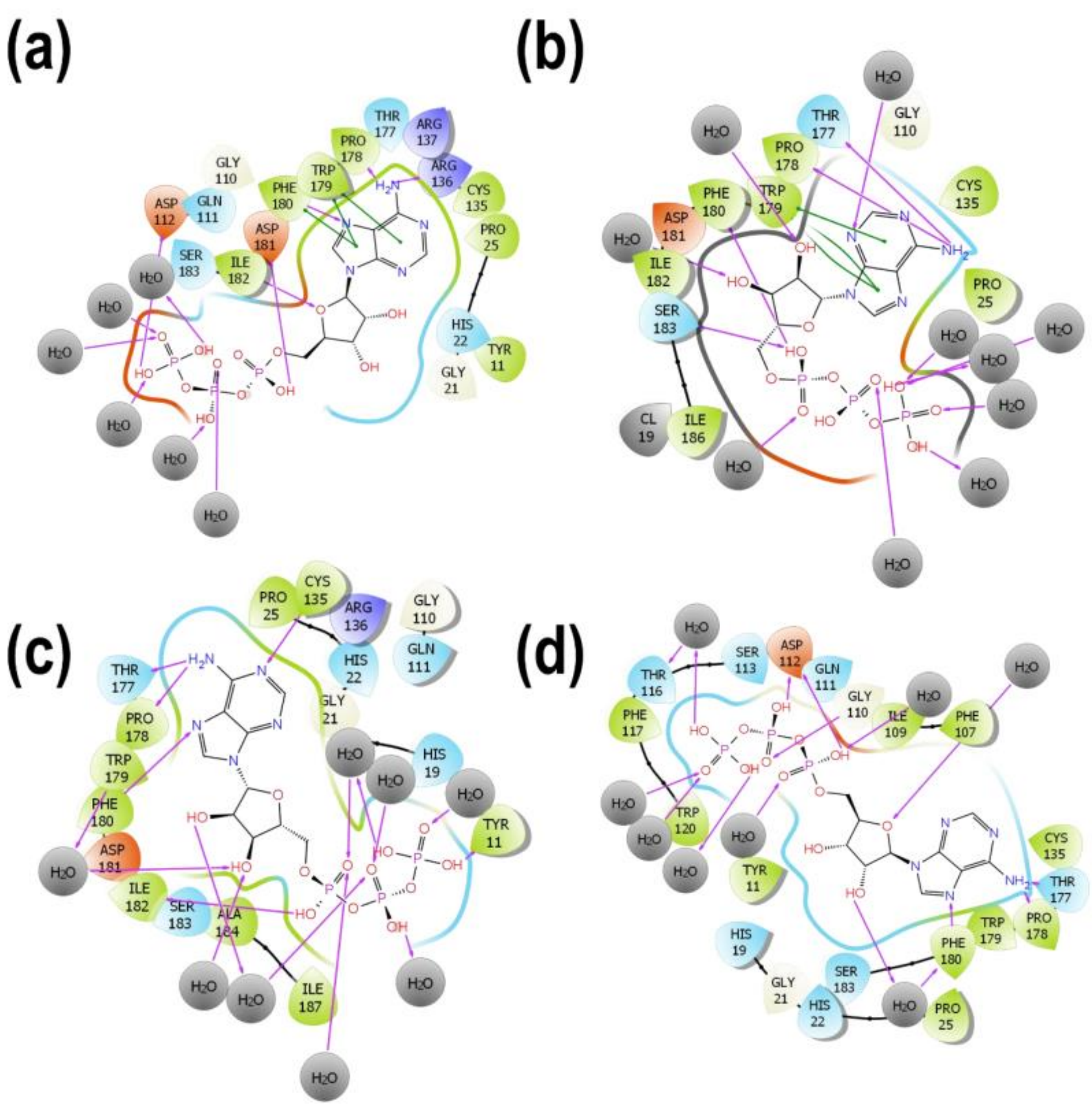

Figure S9. 2D interaction plot showing clusters of trajectory frames based on the RMSD of most dormant screenshots of KpNNAT-ATP complex with (**a**) no metal ion (**b**) Mg^{2+} (**c**) Ca^{2+} and (**d**) Zn^{2+}. The side chain residues are no more than 4 Å from the ligand and are indicated as: blue is polar, green is non-polar, orange is negatively charged, and violet is positively charged. The hydrogen bond is represented as purple lines, salt bridge interactions as blue-red lines, green lines represent π-$\pi*$ interaction, while grey shades indicate exposure to solvent. The images were captured using Maestro 2D interaction diagram.

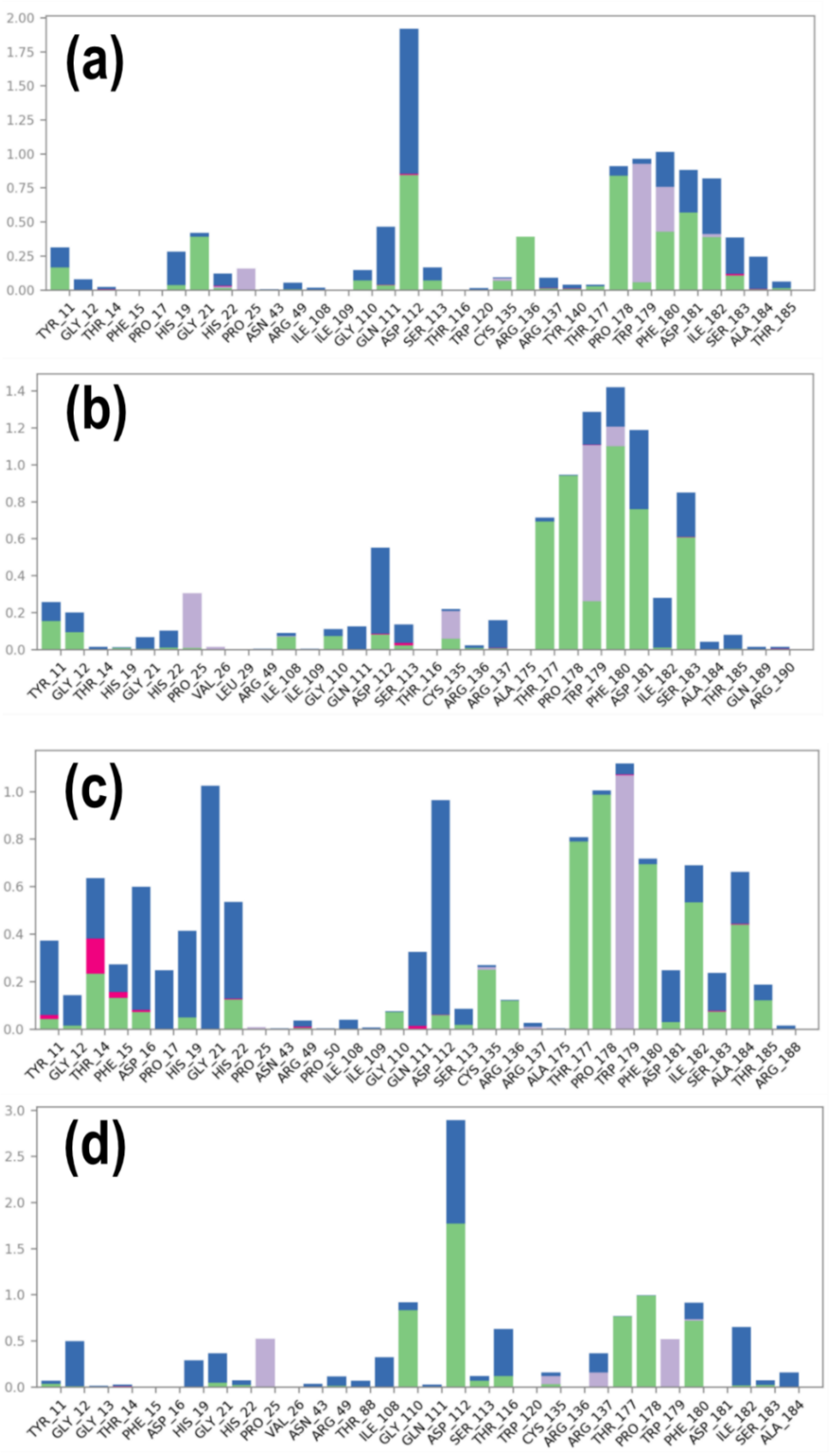

Figure S10. Stacked bar charts of side-chain interactions and the types of interaction between KpNNAT and ATP in (**a**) absence of metal and (**b**) presence of Mg^{2+} (**c**) Ca^{2+} 85 and (**d**) Zn^{2+} over a 100 ns simulation period. 86 The various interactions are represented as Hydrogen bond (green), Hydrophobic interactions (purple) Ionic 87 contacts (pink) and Water bridge (blue). The image was captured via the ligand interaction algorithm imple 88 mented in Maestro v12.2.
